# Transmembrane adaptor protein WBP1L regulates CXCR4 signalling and murine haematopoiesis

**DOI:** 10.1111/jcmm.14895

**Published:** 2019-12-17

**Authors:** Simon Borna, Ales Drobek, Jarmila Kralova, Daniela Glatzova, Iva Splichalova, Matej Fabisik, Jana Pokorna, Tereza Skopcova, Pavla Angelisova, Veronika Kanderova, Julia Starkova, Petr Stanek, Orest V. Matveichuk, Nataliia Pavliuchenko, Katarzyna Kwiatkowska, Majd B. Protty, Michael G. Tomlinson, Meritxell Alberich‐Jorda, Vladimir Korinek, Tomas Brdicka

**Affiliations:** ^1^ Laboratory of Leukocyte Signaling Institute of Molecular Genetics of the Czech Academy of Sciences Prague Czech Republic; ^2^ Faculty of Science Charles University Prague Czech Republic; ^3^ Department of Biophysical Chemistry J. Heyrovsky Institute of Physical Chemistry of the Czech Academy of Sciences Prague Czech Republic; ^4^ Laboratory of Immunobiology Institute of Molecular Genetics of the Czech Academy of Sciences Prague Czech Republic; ^5^ CLIP ‐ Childhood Leukaemia Investigation Prague and Department of Pediatric Hematology and Oncology Second Faculty of Medicine Charles University Prague Czech Republic; ^6^ Second Faculty of Medicine Charles University Prague Czech Republic; ^7^ Laboratory of Molecular Membrane Biology Nencki Institute of Experimental Biology Warsaw Poland; ^8^ Institute of Biomedical Research University of Birmingham Birmingham UK; ^9^ School of Biosciences University of Birmingham Birmingham UK; ^10^ Laboratory of Hematooncology Institute of Molecular Genetics of the Czech Academy of Sciences Prague Czech Republic; ^11^ Laboratory of Cell and Developmental Biology Institute of Molecular Genetics of the Czech Academy of Sciences Prague Czech Republic; ^12^ Present address: Sir Geraint Evans Cardiovascular Research Building Cardiff University Cardiff UK

**Keywords:** bone marrow homing, bone marrow transplantation, CXCR4, ETV6, haematopoiesis, haematopoietic stem cell, NEDD4 family, OPAL1, RUNX1, WBP1L

## Abstract

WW domain binding protein 1‐like (WBP1L), also known as outcome predictor of acute leukaemia 1 (OPAL1), is a transmembrane adaptor protein, expression of which correlates with *ETV6‐RUNX1* (t(12;21)(p13;q22)) translocation and favourable prognosis in childhood leukaemia. It has a broad expression pattern in haematopoietic and in non‐haematopoietic cells. However, its physiological function has been unknown. Here, we show that WBP1L negatively regulates signalling through a critical chemokine receptor CXCR4 in multiple leucocyte subsets and cell lines. We also show that WBP1L interacts with NEDD4‐family ubiquitin ligases and regulates CXCR4 ubiquitination and expression. Moreover, analysis of *Wbp1l*‐deficient mice revealed alterations in B cell development and enhanced efficiency of bone marrow cell transplantation. Collectively, our data show that WBP1L is a novel regulator of CXCR4 signalling and haematopoiesis.

## 1 INTRODUCTION

WW domain binding protein 1‐like (WBP1L) also known as outcome predictor of acute leukaemia 1 (OPAL1) has attracted attention because of a report showing that its elevated expression at mRNA level correlates with favourable outcome in childhood acute lymphoblastic leukaemia (ALL).^1^ These data suggested that it could potentially serve as a prognostic marker. Later, it was shown that its levels are particularly increased in B cell progenitor ALL (BCP‐ALL) with chromosomal translocation t(12;21)(p13;q22), which results in expression of *ETV6‐RUNX1* fusion transcription factor.^2^, ^3^ In BCP‐ALL, this translocation is associated with good prognosis, which likely explains the correlation between WBP1L expression and favourable outcome.^2^ However, it is not known whether WBP1L functionally contributes to it.

ETV6, a fusion partner in *ETV6‐RUNX1*, is a transcriptional repressor and *WBP1L* is one of its target genes.^4^, ^5^, ^6^ In general, ETV6 targets are of high interest because of critical importance of ETV6 in haematopoiesis and its involvement in leukaemia. Around 30 fusions of *ETV6* to different partner genes and a number of mutations in *ETV6* have been identified so far, many of them implicated in various haematological malignancies of myeloid and lymphoid origin.^7^, ^8^ In addition, its critical role in normal haematopoiesis has been revealed in studies of ETV6‐deficient mice, which show profound defects in haematopoietic stem and progenitor cell function and inability of these cells to reconstitute haematopoiesis after bone marrow transplantation.^9^, ^10^

Bioinformatic sequence analysis revealed that WBP1L is a transmembrane adaptor protein with a very short extracellular/luminal part followed by a single transmembrane domain and a larger cytoplasmic tail.^11^ Although relatively short, the extracellular/luminal part presumably forms a small compact domain held together by disulphide bridges formed among cysteines in the C*C*CC*CC motif.^11^ The cytoplasmic part of WBP1L contains several potential interaction motifs corresponding to the consensus sequence of WW domain binding motifs L‐P‐X‐Y or P‐P‐X‐Y.^11^

Except for the limited bioinformatics analysis, WBP1L protein remained completely uncharacterized. Its physiological function has been unknown and whether it has any functional features that may link it to normal haematopoiesis or neoplasia has never been investigated. Here, we show that it binds several members of the NEDD4‐family of ubiquitin ligases and that its deficiency results in enhanced surface expression and signalling of critical chemokine receptor CXCR4. WBP1L deficiency also results in alterations in B cell development and altered dynamics of stem and progenitor cells in the bone marrow. Taken together, we establish the role of WBP1L in CXCR4 signalling and in normal haematopoiesis. These findings also form the basis for further research on its potential role in leukaemia.

## 2 MATERIALS AND METHODS

### 2.1 Protein isolation, detection and quantification assays

Immunoprecipitations (IP) and immunoblotting were performed essentially as reported with adjustments described in online supplement. Western blot quantifications were carried out using Azure c300 imaging system (Azure Biosystems) and Aida Image Analysis software (Elysia‐raytest). WBP1L expression in B cell lines was analysed by size exclusion chromatography‐microsphere‐based affinity proteomics analysis described in detail here,^3^ and the data were quantified using Matlab (MathWorks). Tandem purification of WBP1L was based on the following publication^12^ with modifications described in online supplement. WBP1L palmitoylation was analysed using metabolic labelling with palmitic acid analogue 17ODYA followed by reaction with biotin‐azide and enrichment on streptavidin‐coupled beads as described in detail in online supplement.

### 2.2 Antibodies

Antibodies are listed in Tables S1 and S2. WBP1L antisera were generated by immunization of rabbits with KLH‐conjugated peptide from WBP1L C‐terminus while WBP1L monoclonal antibodies were prepared by standard hybridoma technology after immunization of mice with recombinant C‐terminal part of murine WBP1L protein as described in online supplement.

### 2.3 Cloning, qPCR, DNA transfection, virus preparation and cell infection

cDNA was generated using Quick‐RNA kit (Zymo Research), revert aid reverse transcriptase (Thermo‐Fisher) and oligo‐dT primer. qPCR reactions were run on LightCycler 480 Instrument II using LightCycler 480 SYBR Green I Master mix (Roche). List of qPCR primers is in Table S3. For construct preparation see online supplement and Table S4. Phoenix cell transfection, virus generation and cell transduction were performed as described.^13^ For lentivirus production, the procedure was to a minor extent modified as described in online supplement. Infected cells were sorted on Influx (BD) or selected on G418 (Thermo‐Fisher).

### 2.4 Mouse experiments, homing assays

*Wbp1l* ^−/−^ mice (*Wbp1l^tm2a(EUCOMM)Hmgu^*) on C57Bl/6J genetic background were obtained from International Mouse Phenotyping Consortium. In these mice, gene trap flanked by FRT sites followed by coding region of exon 5 surrounded by LoxP sites were inserted into *Wbp1l* locus by homologous recombination (*Wbp1l* ^−/−^). Mice were bred in specific pathogen free conditions. To obtain inducible *Wbp1l^fl/fl^CreERT* mice, we crossed animals of the *Wbp1l* ^−/−^ strain to *B6.Cg‐Tg (ACTFLPe)9205 Dym/J* mice to remove the gene trap, and subsequently, to *B6.129‐Gt(ROSA)26Sor^tm1(cre/ERT2)Tyj^/J* animals. Both mouse strains were purchased from the Jackson Laboratory (Bar Harbor). To achieve *Wbp1l* deletion, mice were injected intraperitoneally with five daily doses of 2 mg of tamoxifen (Merck) in corn oil (Merck). For homing and transplantation assays, congenic *Ly5.1 *(*C57BL/6NCr*) or *Ly5.1/Ly5.2 *(*C57Bl/6J*) heterozygote recipients were sublethally (three Grey) or lethally (seven Grey) irradiated, followed by injection of transplanted cells into the tail vain. For experiments we used 8‐ to 12–week‐old sex and age matched animals. Housing of mice and in vivo experiments were performed in compliance with local legal requirements and ethical guidelines. The Animal Care and Use Committee of the Institute of Molecular Genetics approved animal care and experimental procedures (Ref. No. 69/2014, 6/2016).

### 2.5 Transwell migration

1 × 10^5^ Cells in DMEM with 0.2% BSA were plated in the upper well of 5µm pore Transwell apparatus (Corning). After 4 hours, migrated cells in the bottom well were counted using a flow cytometer (LSRII; BD).

### 2.6 Statistics

Results represent means ± SEM. If not specified otherwise, *P*‐values were calculated using two‐tailed Student's *t* test, one‐way ANOVA or *Q* test. N represents number of animals or values per group or number of independent experiments.

## 3 RESULTS

### 3.1 WBP1L is a palmitoylated glycoprotein broadly expressed in haematopoietic cells

**Figure 1:**
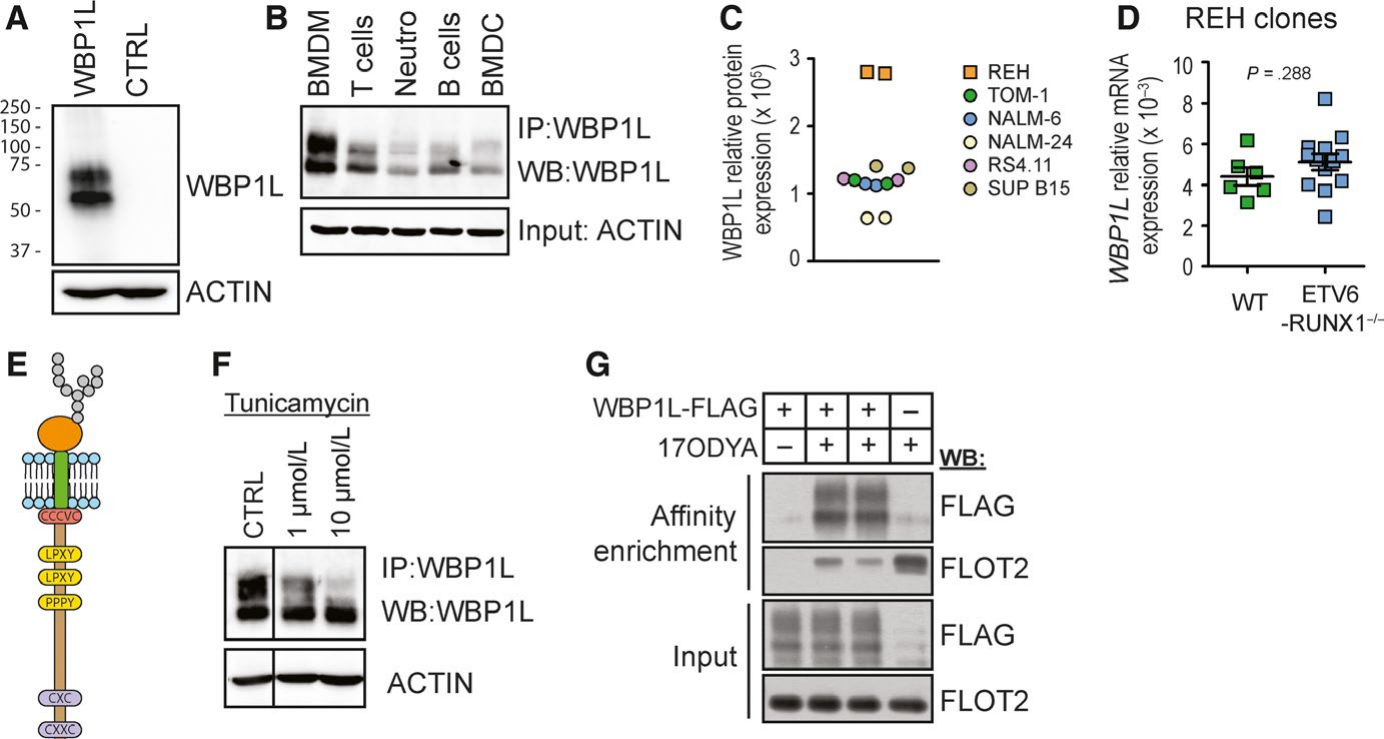
WBP1L is a palmitoylated glycoprotein, broadly expressed in haematopoietic cells. (A) Verification of new WBP1L rabbit antisera specificity on the lysates of HEK293 cells transfected or not with WBP1L construct. (B) Western blot analysis of WBP1L expression in murine leucocyte subsets. T cells (CD3^+^), B cells (CD43^−^, CD11b^−^) and neutrophils (Ly6G^+^) were isolated from the spleen or bone marrow. Bone marrow‐derived macrophages (BMDM) and bone marrow‐derived dendritic cells (BMDC) were differentiated in vitro from murine bone marrow. N = 3. (C) Expression of WBP1L in BCP‐ALL cell lines was probed using size exclusion chromatography‐microsphere‐based affinity Proteomics method. Expression in *ETV6‐RUNX1* ^+^ B cell line REH and *ETV6‐RUNX1* ^−^ lines TOM‐1, NALM‐6, NALM‐24, RS4.11 and SUB B15 was probed by two antibody clones to WBP1L (OPAL‐01, OPAL‐02) and quantified as an area under the curve on parts of chromatograms representing fractions corresponding to WBP1L (N = 1 per antibody clone). (D) Expression of *WBP1L* mRNA in different *ETV6‐RUNX1* ^−/−^ REH clones from two independent sources of REH cells. Data are plotted as 2^‐Δc^ *^t^* (N = 3). (E) Schematic representation of WBP1L structure and conserved sequence motifs (F) Analysis of WBP1L glycosylation in BMDM. BMDM treated or not with 1 or 10 µmol/L tunicamycin (overnight) were subjected to WBP1L immunoprecipitation followed by immunoblotting with WBP1L antisera. β‐actin was stained in the corresponding cell lysates (N = 2). Irrelevant lines from the blot image were removed and replaced with a vertical dividing line. (G) Analysis of palmitoylation of WBP1L. HEK293 cells expressing WBP1L‐FLAG‐STREP were metabolically labelled with palmitate analogue 17ODYA and lysed. 17ODYA labelled proteins were tagged in a click chemistry reaction with biotin‐azide, purified on streptavidin‐coupled beads and analysed for the presence of WBP1L with anti‐FLAG antibody (upper panel) or FLOTILLIN‐2 as a representative of endogenous palmitoylated proteins (middle upper panel). WBP1L expression in cell lysates (middle lower panel) and comparable loading were verified by immunoblotting with antibodies to FLAG‐tag (WBP1L) or FLOTILLIN‐2, respectively (N = 3)

Analysis of WBP1L protein and mRNA expression in murine and human haematopoietic system with a newly generated polyclonal rabbit antibody (Figure 1A) and with Genevestigator tool, respectively, revealed that WBP1L is broadly expressed across multiple human and murine haematopoietic cell subsets (Figure 1B and S1). In addition and in agreement with previous reports of deregulated WBP1L expression in *ETV6‐RUNX1* ^+^ BCP‐ALL, we have found elevated levels of WBP1L protein in REH cell line, which is derived from *ETV6‐RUNX1* ^+^ BCP‐ALL. (Figure 1C and S2A). Interestingly, the genetic deletion of *ETV6‐RUNX1* in REH cells (Figure S2B,C) did not alter WBP1L expression in these cells (Figure 1D).

Imaging of murine bone marrow‐derived macrophages transduced with retroviral vector coding for murine WBP1L fused to EGFP revealed relatively broad distribution of WBP1L‐EGFP within these cells. We have observed co‐localization with plasma membrane, Golgi, endoplasmic reticulum, and to a limited extent with lysosomes and/or other acidic organelles (Figure S3). On the other hand, no co‐localization with mitochondria could be detected (Figure S3).

The N‐terminal part of WBP1L protein is highly conserved among major vertebrate classes (Figure S4). This region contains several conserved motifs, including potential N‐glycosylation (NXS) and palmitoylation sites (Figure 1E and S4). Indeed, we could confirm that WBP1L is both glycosylated and palmitoylated (Figure 1F,G).

### 3.2 WBP1L interacts with NEDD4‐family E3 ubiquitin ligases

**Figure 2:**
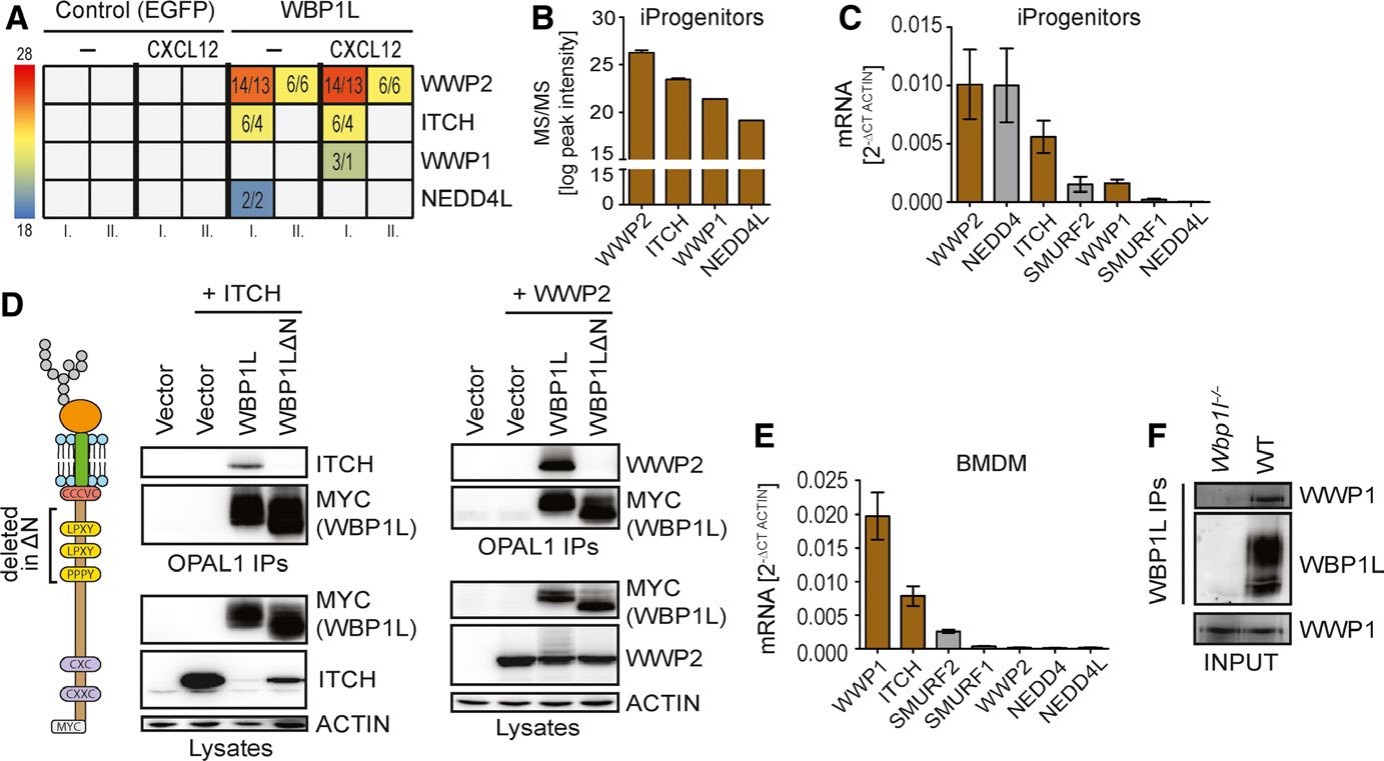
WBP1L binds multiple NEDD4‐family E3 ubiquitin ligases. (A) Data from two independent mass spectrometry analyses (I. and II.) of WBP1L binding partners. *Wbp1l* ^−/−^ monocyte/macrophage progenitors were transduced with the constructs coding for WBP1L‐FLAG‐STREP or EGFP with the same tag. The cells were stimulated for 2 min with CXCL12 or left untreated. Tagged proteins with their binding partners were isolated by tandem purification and subjected to mass spectrometry analysis. The data are presented as colour‐coded intensities obtained by label‐free quantification of NEDD4‐family E3 ubiquitin ligases. Values represent number of peptides used for intensity calculation/ number of unique peptides. Samples, where no peptides from a particular E3 ligase were detected, are coloured in grey (B) Label‐free quantification of interacting E3 ligases from mass spectrometry experiment. Combined average intensities from both CXCR4 stimulated and non‐stimulated samples are plotted (experiment I. form (A) only). (C) mRNA expression of NEDD4‐family E3 ubiquitin ligases in immortalized monocyte/macrophage progenitors (iProgenitors). Plotted in brown are those ligases that interacted with WBP1L in mass spectrometry experiments (N = 3). (D) WBP1L interaction with NEDD4‐family E3 ligases is dependent on WW binding motifs in WBP1L N‐terminus. HEK293 cells were transfected with WBP1L‐Myc or WBP1LΔN‐Myc (with segment containing all WW binding motifs deleted) together with ITCH or WWP2. Following WBP1L immunoprecipitation, the isolated material and the original lysates were immunoblotted with antibody to ITCH or WWP2 and various controls as indicated (N = 3). (E) mRNA expression of NEDD4‐family E3 ligases in BMDM. Plotted in brown are those ligases that interacted with WBP1L in mass spectrometry experiment (N = 3). (F) Endogenous interaction of WBP1L with WWP1 in BMDM. WBP1L immunoprecipitates from WT or *Wbp1l* ^−/−^ BMDM were immunoblotted with antibody to WWP1 and WBP1L. Input lysates were probed with antibody to WWP1 (N = 3). See also Figure S5

Cytoplasmic part of WBP1L contains three WW domain binding motifs ([L/P]PXY) (Figure 1E and S4). It has been speculated that they may interact with WW domains of NEDD4‐family ubiquitin ligases.^11^ However, this family has nine different members. To investigate whether WBP1L interacts with any of these ligases, we have expressed a FLAG‐STREP‐tagged WBP1L construct in immortalized monocyte/macrophage progenitors. We have selected this cell type because of a relatively high level of *Wbpl1* mRNA in myeloid progenitors (Figure S1). We isolated the FALG‐STREP‐tagged construct together with its associated binding partners from the lysates of these cells via a tandem purification on anti‐FLAG and Streptactin beads. Mass spectrometry analysis of the isolated material revealed that WBP1L indeed interacts with several members of NEDD4‐family. In this particular cell type, WWP2 was the most prominent. However, ITCH, WWP1 and NEDD4L could also be detected in one experiment (Figure 2A). Interestingly, the mass spectrometry signal intensities corresponded to the relative expression levels of these NEDD4‐family members in immortalized monocyte/macrophage progenitors (Figure 2B,C). On the other hand, not all NEDD4‐family members expressed in these cells could be co‐isolated with WBP1L. These data suggest a certain level of WBP1L selectivity for individual NEDD4‐family members.

To find out whether NEDD4‐family ligases bind WBP1L via its [L/P]PXY WW domain binding motifs we have co‐expressed the two highest scoring NEDD4‐family ligases from the mass spectrometry experiment, WWP2 or ITCH, with wild‐type WBP1L construct or with mutant WBP1LΔN lacking the membrane‐proximal WW domain binding sequences (Figure 2D) in HEK293 cells. These cells allow for relatively high level of overexpression, which was ideal for reliable identification of NEDD4 family binding sites in WBP1L. Both E3 ligases could be readily co‐immunoprecipitated with wild‐type WBP1L but not with WBP1LΔN (Figure 2D). These data were further confirmed in a similar experiment in J774 macrophage‐like cell line, which expresses relatively high level of ITCH (not shown). The endogenous ITCH could be co‐isolated with wild‐type WBP1L but not with WBP1LΔN from these cells (Figure S5). To confirm the interaction of WBP1L with a NEDD4‐family member at the endogenous protein level, we have selected bone marrow‐derived macrophages (BMDM) which express the highest levels of WBP1L among the cell types we have tested so far (Figure 1B). WWP1, which is the most abundant NEDD4‐family member in this cell type (Figure 2E), could be co‐isolated with WBP1L in this experiment (Figure 2F).

### 3.3 WBP1L regulates ubiquitination and expression level of NEDD4‐family E3 ubiquitin ligases and of CXCR4

**Figure 3:**
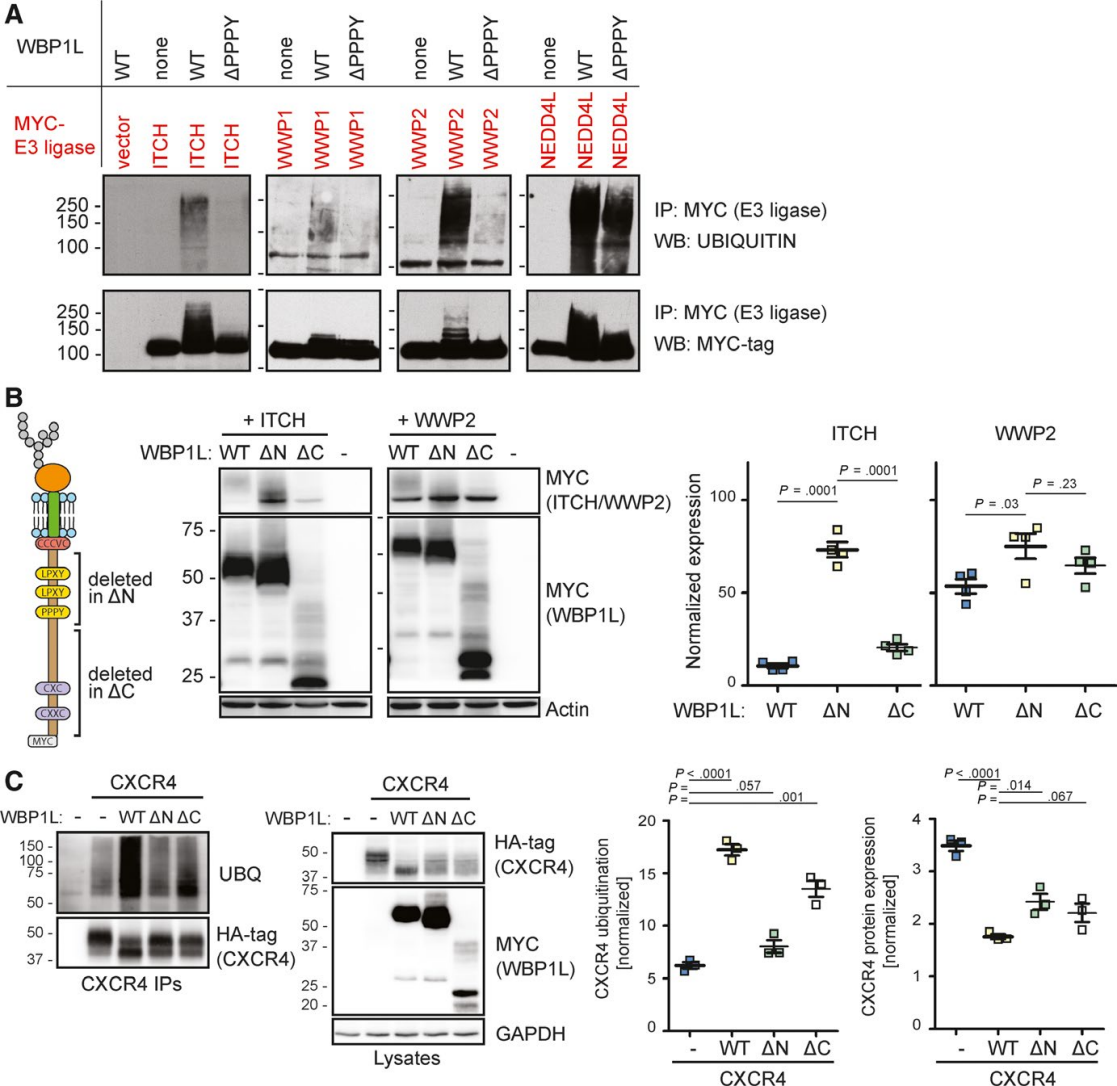
WBP1L regulates ubiquitination and expression of NEDD4‐family E3 ubiquitin ligases and CXCR4. (A) HEK293 cells were cotransfected with individual Myc‐tagged NEDD4‐family E3 ubiquitin ligases and non‐tagged WBP1L or its mutant lacking PPPY WW domain interacting motif. E3 ligases were immunoprecipitated via the Myc‐tag from the lysates of these cells and subjected to immunoblotting with anti‐MYC‐tag or anti‐UBIQUITIN antibody. (B) ITCH and WWP2 stability in HEK293 cells in the presence of WBP1L‐MYC, WBP1L ΔC‐MYC (deletion of almost entire intracellular part of WBP1L except for WW binding motifs) or WBP1L ΔN‐MYC (deletion of WW binding motifs). Lysates from HEK293 cells transfected with ITCH‐MYC or WWP2‐MYC and WBP1L constructs were immunoblotted with antibody to MYC‐tag to visualize ITCH, WWP2 and all forms of WBP1L and with antibody to ACTIN. Quantifications of the data are plotted as values normalized to ACTIN signal and then further normalized to experiment average to allow for comparison among the experiments (N = 3). (C) WBP1L‐mediated increase in CXCR4 ubiqutination and down‐regulation of CXCR4 protein levels in HEK293 cells. WBP1L‐MYC, WBP1LΔC‐MYC or WBP1LΔN‐MYC were cotransfected with CXCR4‐HA followed by CXCR4 imunoprecipitation and immunoblotting with antibodies to ubiquitin or HA‐tag. Lysates were probed with antibodies to HA‐tag (CXCR4), MYC‐tag (WBP1L) or GAPDH (N = 3). For quantification, ubiquitination was normalized to HA‐tag (CXCR4) signal (left panel) and CXCR4 expression to GAPDH (right panel). Both were further normalized to experiment average to allow for comparison among the individual experiments

Interaction with WW domain binding motifs is known to result in the activation and autoubiquitination of NEDD4‐family ubiquitin ligases.^14^, ^15^ To test whether WBP1L can activate NEDD4 family members, we have cotransfected WBP1L‐interacting (Figure 2A) NEDD4‐family ligases with wild‐type WBP1L, or its mutant lacking one of the conserved WW domain binding motifs, into HEK293 cells. This analysis demonstrated that cotransfection with wild‐type but not mutant WBP1L resulted in a substantial increase in ubiquitination of all these ligases, which is a sign of their activation (Figure 3A). Published work suggests that in the case of ITCH this ubiquitination results in down‐regulation of its protein levels, while WWP2 appears relatively resistant to this negative feedback regulation. Thus, to further explore the mechanism of how WBP1L regulates these ubiquitin ligases, we co‐expressed wild‐type WBP1L, WBP1LΔN or WBP1LΔC (lacking C‐terminal region of unknown function) with ITCH or WWP2 in HEK293 cells. Co‐expression of ITCH with wild‐type WBP1L and WBP1LΔC resulted in significantly reduced ITCH protein level when compared to co‐expression with WBP1LΔN. As expected, this effect was much more limited in case of WWP2 (Figure 3B).

One of the best‐studied targets of NEDD4‐family ubiquitin ligases in the haematopoietic system is the chemokine receptor CXCR4. It is involved in the maintenance of haematopoietic stem and progenitor cells and in promoting niche interactions in the bone marrow. It is also thought to support the survival and treatment resistance of leukaemic cells.^16^, ^17^ Based on these features, we have selected CXCR4 for a similar set of experiments to test whether WBP1L regulates its protein expression levels and ubiquitination. Indeed, co‐expression of WBP1L with CXCR4 in HEK293 cells resulted in increased ubiquitination of CXCR4 (presumably by an endogenous NEDD4‐family ligase). This effect was almost completely abolished by mutation of the WW domain binding motifs (WBP1LΔN) while deletion of the C‐terminal sequence (WBP1LΔC) had a more limited effect (Figure 3C). CXCR4 ubiquitination was further accompanied by reduction in CXCR4 protein levels (Figure 3C). We also observed that WBP1L co‐expression resulted in a striking increase in CXCR4 electrophoretic mobility (Figure 3C). The reason for this mobility shift is at present unknown.

### 3.4 WBP1L inhibits CXCR4 signalling in murine and human cell lines

**Figure 4:**
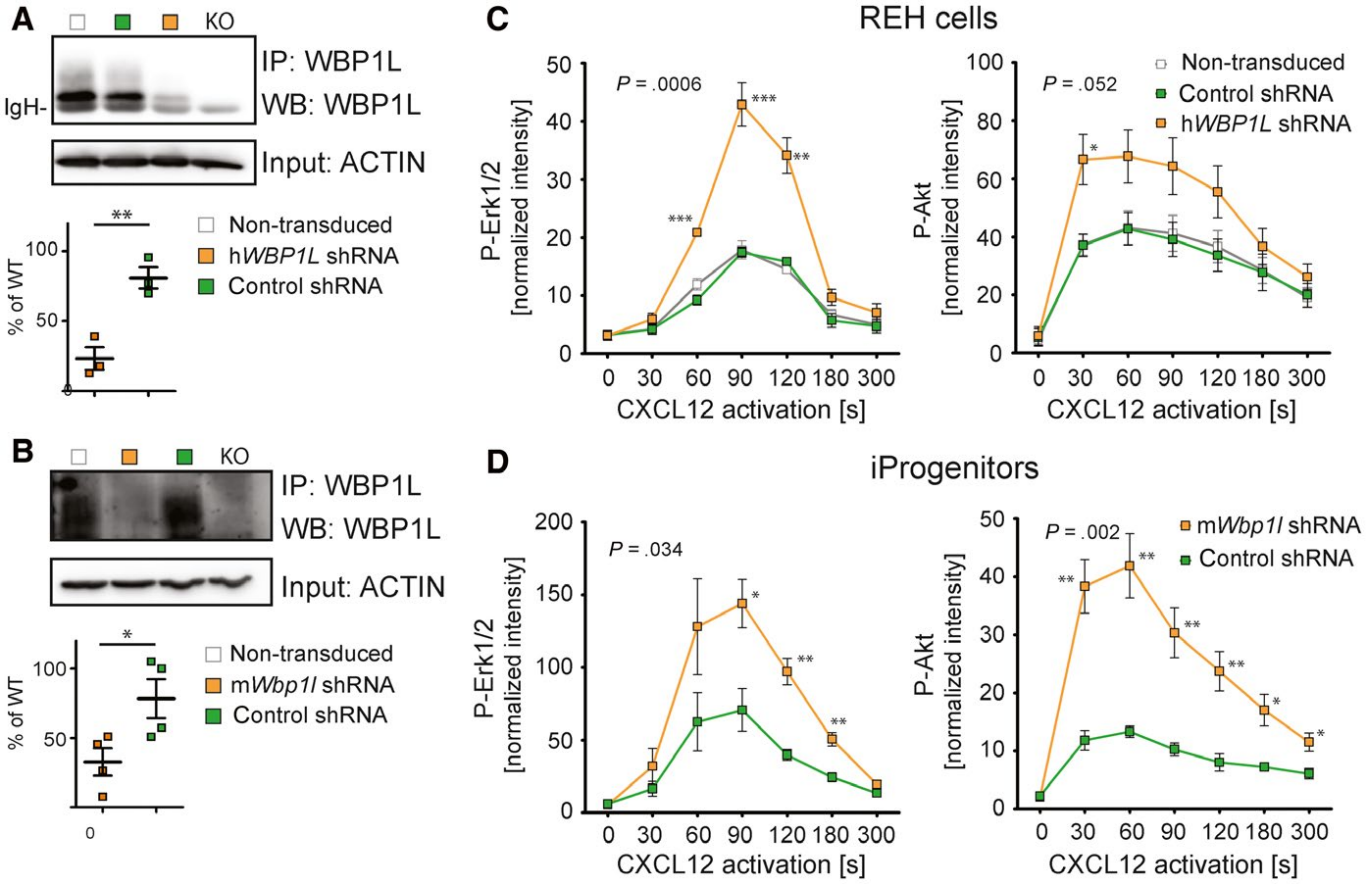
ShRNA‐mediated down‐regulation of WBP1L results in enhanced CXCR4 signalling in human and murine cell lines. (A, B) WBP1L immunoprecipitates from REH cells (A) or immortalized monocyte/macrophage progenitors (B) transduced with *Wbp1l* shRNA were stained with WBP1L antisera to demonstrate WBP1L down‐regulation. Equal input of lysates to immunoprecipitation was verified by ACTIN immunoblotting. Quantification of multiple experiments (after normalization to ACTIN signal) was plotted as a percentage of WBP1L expression in non‐transduced cells. (C, D) ERK1/2 and AKT phosphorylation downstream of CXCR4 in REH cells (C) and immortalized monocyte/macrophage progenitors (iProgenitors) (D), where WBP1L was down‐regulated by shRNA. Cells were stimulated with 100 nmol/L CXCL12, lysed and subjected to Western blot analysis of ERK1/2 and AKT phosphorylation. Data represent mean of fluorescence intensity normalized to GAPDH. The *P*‐value was calculated to compare maximum responses of cells transduced with non‐silencing control and silencing shRNA. Asterisks denote significant differences in individual time‐points. (N = 4)

To analyse the effects of the endogenous WBP1L on CXCR4, we have selected two cell lines where the expression of WBP1L and/or CXCR4 is highly relevant. These included human leukaemic cell line REH as a representative of *ETV6‐RUNX1* ^+^ leukaemia, where we have down‐regulated WBP1L by a single shRNA specific for human *WBP1L* and immortalized murine monocyte/macrophage progenitors as a representative of bone marrow progenitors, where we used a different shRNA targeting murine *Wbp1l* (Figure 4A,B). After stimulation with CXCR4 ligand CXCL12, these cells showed enhanced activity of downstream signalling pathways, resulting in increased phosphorylation of ERK1/2 and AKT (Figure 4C,D and S6). CXCR7, another known receptor for CXCL12, did not contribute to the signalling output under these conditions (Figure S7). These data demonstrated that WBP1L is involved in the negative regulation of CXCR4 signalling.

### 3.5 Altered haematopoiesis in *Wbp1l*‐deficient mice

**Figure 5:**
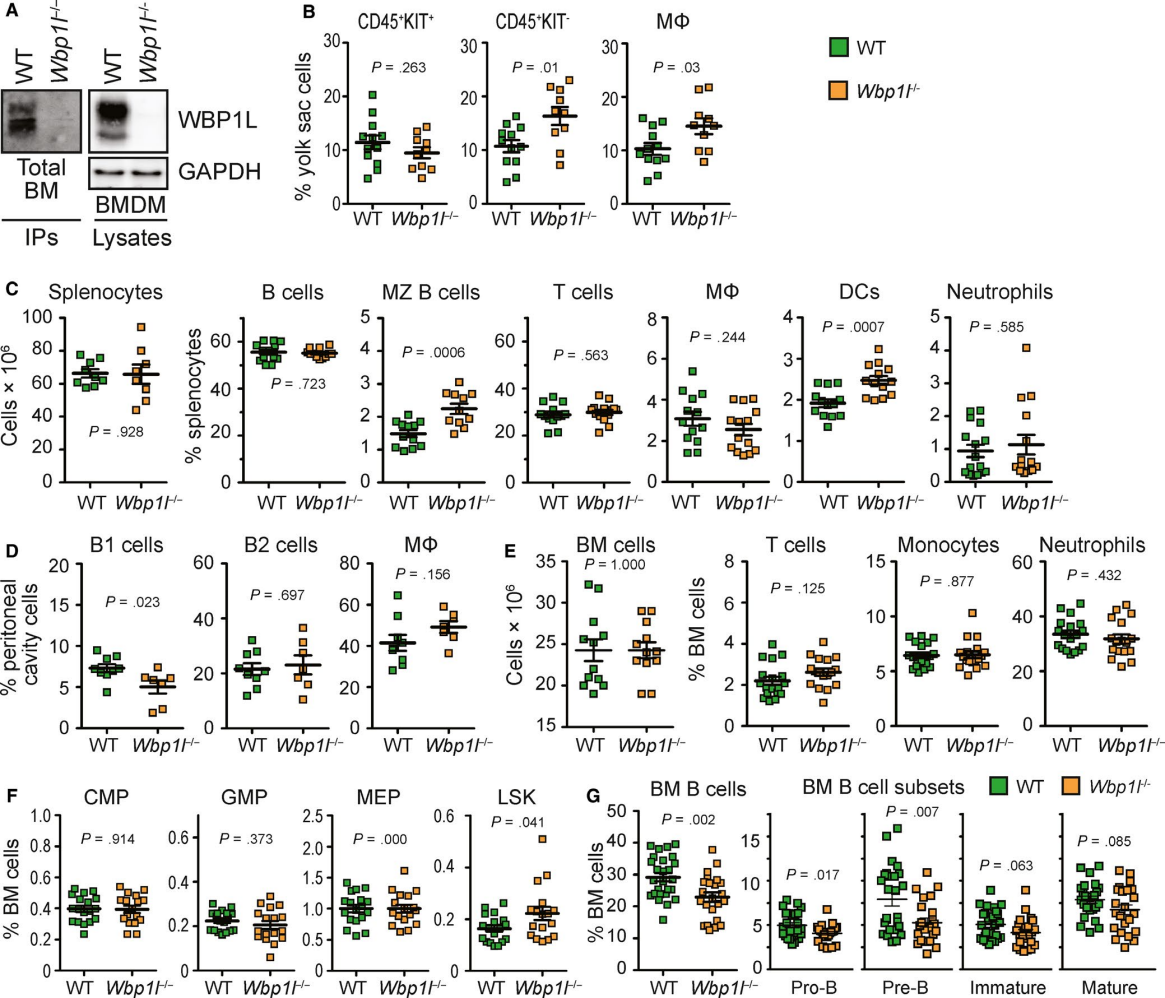
Altered leucocyte homeostasis in WBP1L‐deficient mice. (A) Western blot analysis of WBP1L immunoprecipitates or whole cell lysates prepared from WT and *Wbp1l* ^−/−^ cells. mOPAL‐01/03 antibodies were used for immunoprecipitation and WBP1L rabbit antisera for Western blotting. N = 3 (bone marrow), N = 5 (BMDM). (B) Flow cytometry analyses of E10.5 WT and *Wbp1l* ^−/−^ yolk sac cell subsets. Primitive macrophages were defined as Ter119^−^ CD11b^+^ F4/80^+^. (C) Absolute numbers of splenocytes obtained from WT and *Wbp1l* ^−/−^ mice and flow cytometry analyses of WT and *Wbp1l* ^−/−^ splenocytes defined using the following markers: B cells (B220^+^), MZ B cells (B220^+^, AA4.1^−^, CD23^−/low^, CD1d^+^), T cells (CD3^+^), macrophages (F4/80^+^, CD11b ^int^), DC (CD11c^+^, LY6C^−/low^) and neutrophils (LY6G^+^, CD11b^+^, LY6C^−^). (D) Flow cytometry analyses of WT and *Wbp1l* ^−/−^ leucocytes in the peritoneum, defined using the following markers: B1 cells (SSC^low^, FSC^low^, B220^+^, CD23^−/low^), B2 cells (SSC^low^, FSC^low^, B220^+^, CD23^+^) and macrophages (large peritoneal macrophages, CD11b^high^,F4/80^high^). (E) Flow cytometry analyses of WT and *Wbp1l* ^−/−^ bone marrow cell subsets, defined using the following markers: T cells (CD3^+^), monocytes (LY6C^+^, CD11b^+^, LY6G^−^, CD19^−^, TER119^−^, CD3^−^, NK1.1^−^), neutrophils (KIT^−^, B220^−^, TER119^−^, CD3^−^, LY6G^high^) (F) Flow cytometry analyses of stem and progenitor cells in the bone marrow. Cell subsets were defined using following markers: common myeloid progenitors—CMP (lin^−^, c‐kit^+^, CD34^+^, CD16/32^neg−low^, SCA1^−^), granulocyte‐monocyte progenitors—GMP (lin^−^, KIT^+^, CD34^+^, CD16/32^High^, SCA1^−^), megakaryocyte‐erythroid progenitors—MEP (lin^−^, KIT^+^, CD34^−^, CD16/32^−^, SCA1^−^) and LSK (lin^−^, KIT^+^, SCA1^+^). (G) Flow cytometry analysis of bone marrow B cell subsets. B cells (B220^+^), pro‐B cells (CD43^+^, B220^+^, IgM^−^), pre‐B cells (CD43^−^, B220^low^, IgM^−^), immature B cells (CD43^−^, B220^low^, IgM^+^) and mature B cells (CD43^−^, B220^high^, IgM^+^)

To further analyse the physiological function of WBP1L, we have acquired *Wbp1l*‐deficient mouse strain *Wbp1l^tm2a(EUCOMM)Hmgu^* (hereafter referred to as *Wbp1l* ^−/−^) from the International Mouse Phenotyping Consortium. These mice appeared grossly normal and healthy, were born in normal Mendelian ratios and did not express WBP1L protein (Figure 5A).

To characterize the haematopoietic system in *Wbp1l* ^−/−^ mice, we have analysed major cell subset frequencies in adult mice and in embryos. The embryonic haematopoietic cell numbers were grossly normal with small increases in the yolk sac CD45^+^ KIT^−^ cells and CD11b^+^ F4/80^+^ yolk sac macrophages (Figure 5B). In the peripheral tissues of the adult mice, there was a significant increase in marginal zone B cell fraction in the spleen (Figure 5C) and a reduction in B1 cell percentages in the peritoneal cavity (Figure 5D). We also observed increased frequencies of splenic dendritic cells in *Wbp1l* ^−/−^ mice (Figure 5C). Otherwise, the frequencies of other leucocyte subsets found in peripheral tissues were normal (Figure 5C,D). Bone marrows from *Wbp1l* ^−/−^ animals showed the same cell counts as wild‐type bone marrows (Figure 5E). Most of bone marrow cell subsets were also found in normal numbers, including T cells, monocytes, neutrophils (Figure 5E) and the majority of progenitor populations (Figure 5F). However, there were two notable exceptions. First, the overall B cell percentages in the bone marrow were significantly reduced (Figure 5G). The reduction was most pronounced in early developmental stages (pro‐ and pre‐B cells). At the later stages, including immature and mature B cells a similar trend was observed, but it was outside the threshold for statistical significance (*P*‐values .06 and .08, respectively) (Figure 5G). The cell cycle of B cell progenitors was not substantially changed with the exception of a very small but significant increase in G1 phase pre‐B cells in *Wbp1l* ^−/−^ mice (Figure S8). Second, Lin^−^SCA1^+^KIT^+^ (LSK) cells encompassing early progenitors and stem cells (HSPC) showed slightly but significantly increased percentages in these animals (Figure 5F).

**Figure 6:**
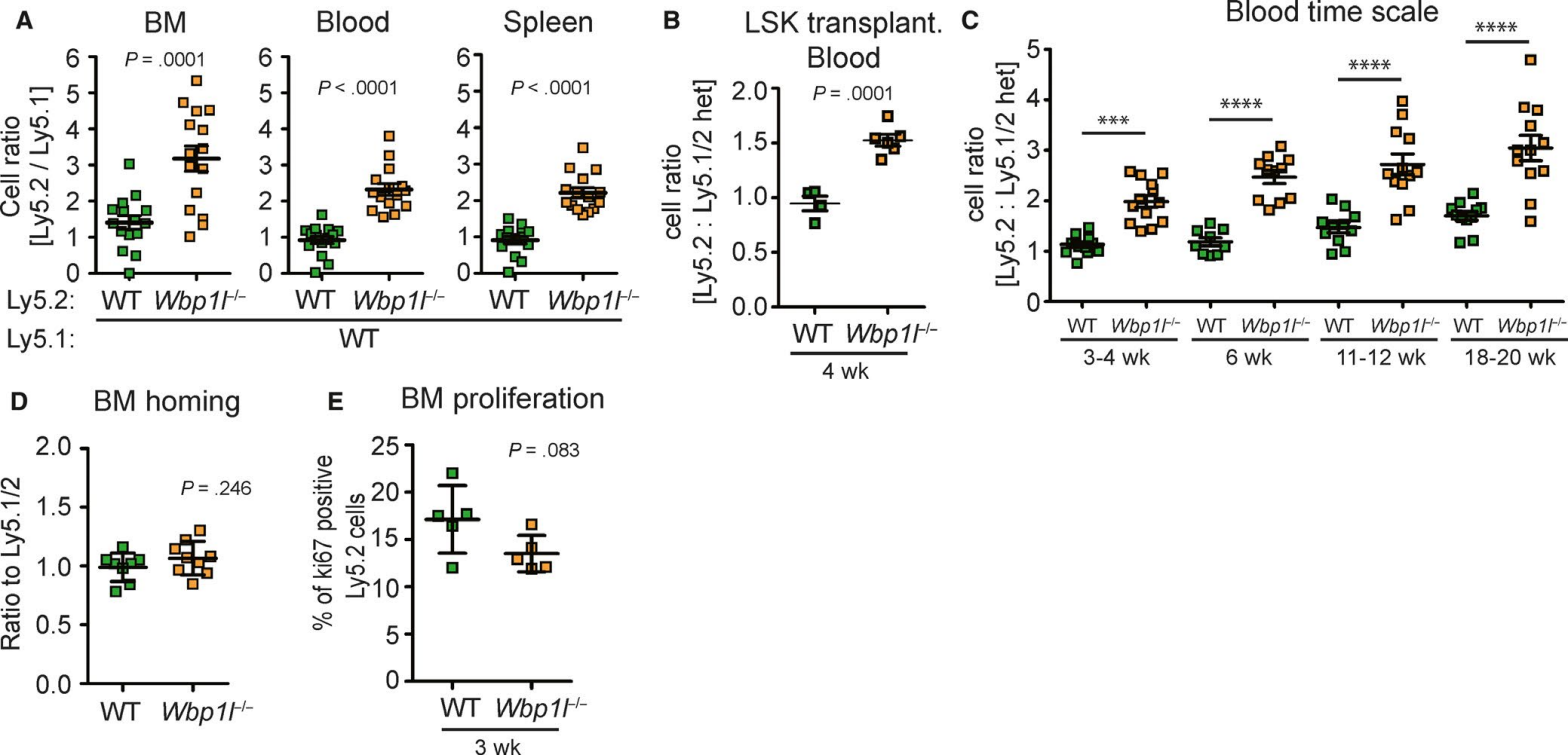
Enhanced engraftment of *Wbp1l* ^−/−^ bone marrow. (A) Ly5.2^+^ bone marrow (WT or *Wbp1l* ^−/−^) was mixed with Ly5.1^+^ bone marrow (always WT) in a ratio 1:1 and 2 × 10^6^ cells were transplanted into Ly5.1 lethally irradiated mice. Mice were analysed 2 months post‐transplantation. Flow cytometry analyses show the ratio between Ly5.2 and Ly5.1 cells in the bone marrow, blood and spleen. (B) LSK cells sorted from Ly5.2^+^ bone marrow (WT or *Wbp1l* ^−/−^) were mixed with LSK from Ly5.1/Ly5.2^+^ heterozygous bone marrow (always WT) in a ratio 1:1 and 20 000 cells together with support of 0.5 × 10^6^ Ly5.1 bone marrow cells were injected into tail vain of Ly5.1 lethally irradiated mice. Data represent the ratio between Ly5.2^+^ and Ly5.1/Ly5.2^+^ cells detected in the recipient blood 4 wk post injection. One of two independent experiments is shown (N ≥ 8). (C) Ly5.2^+^ bone marrow (WT or *Wbp1l* ^−/−^) was mixed with Ly5.1/Ly5.2^+^ heterozygous bone marrow (always WT) in a ratio 1:1 and 2 × 10^6^ cells were transplanted into Ly5.1 lethally irradiated mice. At indicated time‐points, the ratio between Ly5.2 and Ly5.1/Ly5.2 cells in blood was measured by flow cytometry. (D) Homing of WT and *Wbp1l* ^−/−^ bone marrow cells in a competitive set‐up. WT or *Wbp1l* ^−/−^ bone marrow (Ly5.2^+^) was each combined with WT bone marrow from Ly5.1/Ly5.2^+^ heterozygotes in a ratio 1:1 and 2 × 10^6^ cells were injected into the tail vain of sublethally irradiated recipient (Ly5.1^+^). Data represent the ratio between Ly5.2^+^ and Ly5.1/Ly5.2^+^ cells detected in the recipient bone marrow 16 h post injection (N ≥ 8). (E) Competitive bone marrow transplantation was performed as in (C) and 3 wk after the transplantation expression of Ki67 proliferation marker was measured in transplanted cells

To test their functionality in vivo, we performed a competitive bone marrow transplantation assay, whereby we mixed wild‐type or *Wbp1l* ^−/−^ Ly5.2 cells with wild‐type Ly5.1 bone marrow cells in a 1:1 ratio and transplanted these mixtures into lethally irradiated recipient mice (Ly5.1). Nine weeks later, we have analysed their engraftment. Strikingly, *Wbp1l* ^−/−^ bone marrow engrafted significantly better and the ratio between *Wbp1l* ^−/−^ and wild‐type cells increased from 1:1 to ca 3:1 (Figure 6A), whereas wild‐type Ly5.2 and Ly5.1 BM engrafted with equal efficiency. The difference could be observed across all bone marrow leucocyte subsets analysed except for LSK cells, where a similar trend in favour of *Wbp1l* ^−/−^ cells was also present but was not statistically significant (Figure S9). The difference was also maintained in the periphery, where the ratio between *Wbp1l* ^−/−^ and wild‐type cells was roughly 2:1 (Figure 6A). Similar difference in engraftment efficiency was also observed when we transplanted sorted LSK cells in the 1:1 ratio (Figure 6B). Next, we investigated how this ratio changes with time. A significant difference between the engraftment efficiency could be observed as early as 3 weeks after the transplantation and was maintained till at least 18 weeks after the transplantation (Figure 6C). The increased efficiency in the bone marrow engraftment was not the result of increased homing to the bone marrow or increased proliferation, which did not display any alteration (Figure 6D,E). Collectively, these data are showing negative role of WBP1L in HSPC function. Persistence of the engraftment advantage for more than 16 weeks suggests that the haematopoietic system is affected already at the level of haematopoietic stem cells.

### 3.6 Compensatory mechanisms restore CXCR4 signalling when WBP1L is lost in the germline, but the effects of WBP1L deficiency on CXCR4 signalling can be observed upon its acute deletion

**Figure 7:**
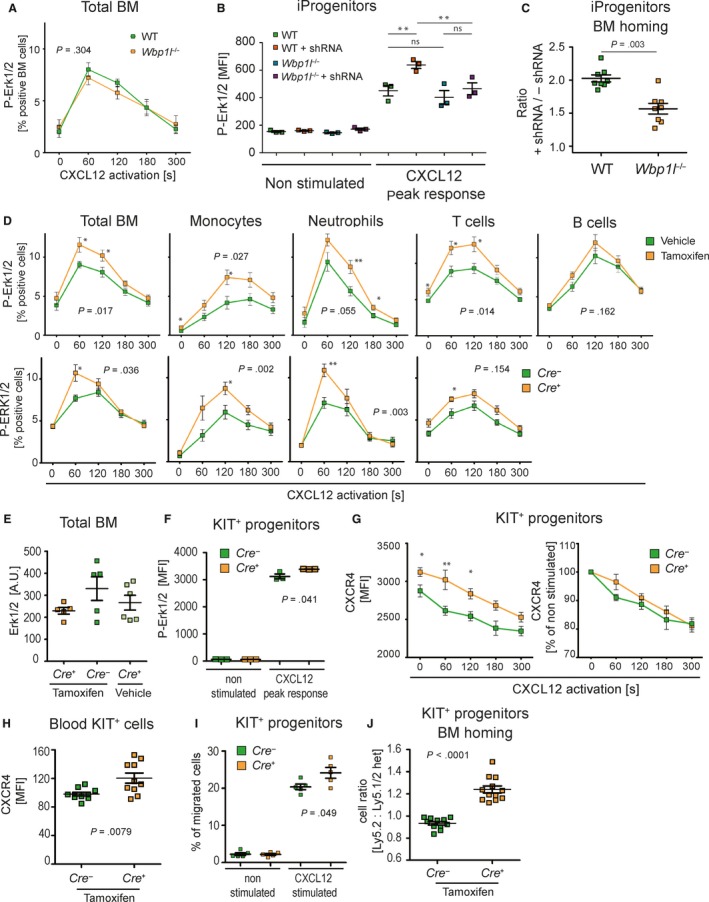
Acute loss of WBP1L results in enhanced CXCR4 signalling but germline deficiency is compensated for. (A) ERK1/2 phosphorylation downstream of CXCR4 in WT and *Wbp1l* ^−/−^ (germline deletion) bone marrow cells. Cells were stimulated with 100 nmol/L CXCL12, fixed, stained for phosphorylated ERK1/2 and analysed by flow cytometry. Data represent percentage of responding cells (N = 6). *P*‐value was calculated for maximum response of WT and *Wbp1l* ^−/−^ cells regardless of the time‐point where it was reached. (B) Erk1/2 activation after CXCL12 stimulation (100 nmol/L) of immortalized monocyte/macrophage progenitors (iProgenitors) from WT and *Wbp1l* ^−/−^ mice and of the same cells transduced with *Wbp1l* shRNA. Erk phosphorylation was measured by flow cytometry of fixed and permeabilized cells stained with fluorescent P‐Erk1/2 antibody. Peak response detected during 5 min measurement is shown. Data are represented as medians of fluorescence intensity (N = 3). *P*‐values were calculated using 1way ANOVA with Bonferroni's multiple comparison test. (C) WT cells transduced or not with *Wbp1l* shRNA were mixed 1:1 and their bone marrow homing ability was analysed as in Figure 6D. As a control *Wbp1l* ^−/−^ cells transduced or not with *Wbp1l* shRNA and also mixed in a ratio 1:1 were used. Ratios of shRNA transduced and non‐transduced cells are plotted for each genotype (n = 8). *P*‐value was calculated for the differences between these two ratios. Significant outliers were discarded based on *q* test. As not all cells expressed shRNA containing vector after transduction, the true ratio was lower than 1:1 at the time of injection. The final data were normalized to this true ratio. (D) CXCR4 signalling in bone marrow cell subsets after acute deletion of *Wbp1l*. In the top row, *CreERT* expressing cells treated either with tamoxifen or vehicle (corn oil) are compared. In the bottom row, the comparison is made between tamoxifen‐treated *Wbp1l^fl/fl^‐CreERT* and *Wbp1l^fl/fl^* (without *CreERT*) bone marrow cells. Cells were stimulated with 100 nmol/L CXCL12, fixed, stained for extracellular markers and intracellular P‐ERK and analysed by flow cytometry. Data represent percentage of responding cells from whole bone marrow (N = 7), T cells (CD3^+^, N = 7/8), monocytes (Ly6C^+^ Ly6G^−^, N = 11) and neutrophils (Ly6G^+^ Ly6C^−^,N = 7). Significant outliers were discarded based on *q* test. *P*‐value was calculated for maximum response (regardless of the time‐point at which this maximum was reached). In addition, significant differences in individual time‐points are labelled with asterisks. (E) Expression of total ERK1/2 in the bone marrow cells where *Wbp1l* was deleted as in (D). Protein level was measured using Western blot. ERK1 and ERK2 were probed separately and signal was summed and normalized to actin loading control (N ≥ 5) (F). CXCR4 signalling in in vitro 4‐hydroxytamoxifen treated KIT^+^ progenitors isolated from *Wbp1l^fl/fl^‐CreERT* and *Wbp1l^fl/fl^* (without *CreERT*) bone marrow. ERK phosphorylation was measured after stimulation with 100 nmol/L CXCL12 by flow cytometry on fixed and permeabilized cells. Peak response detected during 5 min measurement is shown. One of two independent experiments is shown, N = 6. (G) CXCL12‐induced changes of CXCR4 surface expression on KIT^+^ bone marrow progenitors. These cells were isolated from *Wbp1l^fl/fl^‐CreERT *and *Wbp1l^fl/fl^* bone marrow, treated with 4‐hydroxytamoxifen to induce *Wbp1l* deletion and stimulated with CXCL12 for indicated time intervals. FACS data are plotted as median fluorescence intensities (left graph) or as percentage of expression level on non‐stimulated cells (right graph) (n = 7). (H) CXCR4 expression on KIT^+^ progenitors detected in the blood of tamoxifen‐treated *Wbp1l^fl/fl^‐CreERT* and *Wbp1l^fl/fl^* mice. Significant outlier was discarded based on *q* test (N = 10). (I) Migration of 4‐hydroxytamoxifen‐treated KIT^+^ progenitors from *Wbp1l^fl/fl^‐CreERT* and *Wbp1l^fl/fl^* mice towards 500 nmol/L CXCR4 in a transwell assay in vitro (N = 5). (J) Ly5.2^+^ KIT^+^ progenitors (from *Wbp1l^fl/fl^‐CreERT* or *Wbp1l^fl/fl^* mice, treated with 4‐hydroxytamoxifen) were mixed 1:1 with Ly5.1/Ly5.2^+^ heterozygous KIT^+^ progenitors from WT mice and 10^7^ cells were injected into Ly5.1 sublethally irradiated recipients. After 16 h, the ratio between Ly5.2 and Ly5.1/Ly5.2 cells in the bone marrow was measured by flow cytometry (N ≥ 11)

Part of the data described above are consistent with CXCR4 hyperactivity. However, the same homing capacity of the wild‐type and *Wbp1l* ^−/−^ bone marrow cells (Figure 6D) is incompatible with enhanced CXCR4 function. These results prompted us to test whether bone marrow cells from *Wbp1l* ^−/−^ mice display similar CXCR4 dysregulation as shRNA‐treated cell lines. Surprisingly, we did not observe any alterations in CXCL12‐triggered ERK phosphorylation in bone marrow cells from *Wbp1l* ^−/−^ mice (Figure 7A). This result was in disagreement with our analysis of the effects of shRNA‐mediated WBP1L down‐regulation in cell lines (Figure 4). To exclude the possibility that enhanced CXCR4 signalling observed there was the result of non‐specific off‐target effects of *Wbp1l* shRNAs, we expressed shRNA targeting *Wbp1l* in immortalized monocyte/macrophage progenitors from wild‐type and *Wbp1l* ^−/−^ mice. Because of the absence of WBP1L, only non‐specific activity of *Wbp1l* shRNA can be detected in *Wbp1l* ^−/−^ progenitors. As expected, *Wbp1l* shRNA significantly enhanced CXCL12‐triggered ERK activation in wild‐type cells. On the other hand, only negligible insignificant changes were detected in *Wbp1l* ^−/−^ cells (Figure 7B). These results demonstrated that the effects of *Wbp1l* shRNA are dependent on *Wbp1l* and, thus, specific. When we used the same cells in an in vivo homing experiment, we observed that WBP1L down‐regulation significantly enhanced bone marrow homing of wild‐type cells, when compared to *Wbp1l* ^−/−^ cells (Figure 7C). This outcome is consistent with the results of our in vitro analyses showing that WBP1L negatively regulates activity of CXCR4.

To definitely prove the validity of our shRNA data, we have generated a mouse strain *Wbp1l^fl/fl^‐CreERT* where the *Wbp1l* gene can be acutely inactivated upon injection of 4OH‐tamoxifen. Acute *Wbp1l* deletion in this model resulted in enhanced ERK phosphorylation in response to CXCL12 stimulation in total bone marrow as well as in several major subsets, including T cells, monocytes and neutrophils, whereas in B cells, the difference was small and not statistically significant (Figure 7D and S10). No effects of WBP1L deficiency on total ERK expression were observed (Figure 7E). The enhanced CXCR4 signalling did not translate to any immediate effect on steady‐state numbers of major bone marrow and splenic cell subsets (Figure S11A). However, it is likely that only much more substantial increase in CXCR4 signalling would be needed to alter leucocyte bone marrow retention within this timescale.

The data presented above suggested that the effects of WBP1L down‐regulation can only be observed after its acute deletion. To further address this possibility, we have established primary culture of isolated KIT^+^ bone marrow cells from *Wbp1l^fl/fl^‐CreERT* mice, where *Wbp1l* could be deleted by 4OH‐tamoxifen. Importantly, cells cultured outside the bone marrow are not exposed to continuous CXCL12 stimulation and desensitization and are, thus, having high expression of CXCR4 and stronger signalling capacity. In agreement with our previous observations, after acute *Wbp1l* deletion by CRE recombinase (but not after germline deletion), these progenitors showed increased ERK phosphorylation in response to CXCL12 stimulation (Figure 7F and S11B). *Wbp1l* deletion also resulted in an increase in steady‐state CXCR4 surface expression with no other effects on the rate of CXCL12‐triggered receptor internalization in these cells (Figure 7G and S11C). The same increase in CXCR4 expression was also observed on KIT^+^ progenitors from peripheral blood of animals after acute *Wbp1l* deletion (Figure 7H). Similar, though not significant, trend was also observed when CXCR4 was measured on fixed and permeabilized cells (Figure S11D). Finally, cells with acute *Wbp1l* deletion showed increased migration towards CXCR4 ligand CXCL12 in a transwell assay in vitro (Figure 7I) and increased bone marrow homing efficiency in vivo (Figure 7J). These data confirm that WBP1L negatively regulates CXCR4 expression and signalling in primary cells.

## 4 DISCUSSION

Expression of the *WBP1L* gene is heightened in *ETV6‐RUNX1* ^+^ paediatric BCP‐ALL and was shown to correlate with favourable treatment response.^1^, ^2^, ^3^ However, the function of its protein product WBP1L in healthy and leukaemic cells has not been investigated. Here, we attempted to uncover its physiological function. Our initial analysis shows that WBP1L binds several NEDD4‐family E3 ubiquitin ligases and its deficiency results in augmented surface expression and signalling of CXCR4, one of their known target proteins. At the organismal level, WBP1L deficiency resulted in perturbations in B cell development and increased ability of bone marrow stem and progenitor cells to reconstitute haematopoietic system after the bone marrow transplantation. In addition, acute *Wbp1l* deletion resulted in increased progenitor homing to the bone marrow. How much of this phenotype can be attributed to CXCR4 hyperactivity is still an open question. There are at least two other mouse models that show increased CXCR4 activity. One of these strains carries a mutation in CXCR4 that prevents its desensitization and down‐regulation (*CXCR4^WHIM^*). The same mutation in humans causes immunodeficiency known as WHIM syndrome.^18^, ^19^, ^20^ *CXCR4^WHIM^* mice display a similar selective dysregulation in the bone marrow B cell compartment as *Wbp1l* ^−/−^ mice with the reduction in B cell percentages that is most profound at the B cell progenitor level.^20^ They also show increase in marginal zone B cell percentages. On the other hand, some of their symptoms were not detected in *Wbp1l* ^−/−^ mice, including blood neutropenia and lymphopenia and reduced spleen size.^20^ Increased CXCR4 expression and CXCR4‐mediated signalling were also observed in mice deficient in the expression of BAR domain containing adaptor protein Missing In Metastasis (MIM). In these animals, leucocyte development and percentages appeared largely normal and no leukopenia has been observed. Rather they showed slightly increased white blood cell counts and splenomegaly.^21^, ^22^ Even though MIM deficiency has a number of CXCR4‐independent effects, these data show that enhanced CXCR4 activity does not have to lead to leukopenia in the peripheral tissues, nor to major alterations in leucocyte subset numbers and frequencies in the bone marrow. It is possible that the lack of receptor desensitization rather than alterations in peak signal intensity may be the key factor driving peripheral leukopenia in *CXCR4^WHIM^* mice.

Since in mice with germline *Wbp1l* deletion we have not observed up‐regulation in CXCR4 activity, it is difficult to unequivocally answer the question if alterations in *Wbp1l* ^−/−^ B cell development are caused by CXCR4 dysregulation. This phenotype is strikingly similar to the one observed in *CXCR4^WHIM^* animals. It is possible that not all haematopoietic cell subsets are able to compensate for the loss of WBP1L. Early B cell progenitors represent a relatively small population, and their WBP1L expression based on the data from ImmGen consortium^23^ is similar to other B cell subsets (not shown). The options to analyse their CXCR4 signalling pathways are relatively limited. Those that can be analysed by flow cytometry, including ERK and STAT3 phosphorylation, as well as calcium response appear to be hypo/non‐responsive in this particular cell type despite clearly measurable CXCR4 expression (not shown). As a result, B cell progenitors contribute very little to ERK phosphorylation of the entire B cell pool measured in our experiments where the more mature stages dominated the response. The other pathways we were not able to analyse in this relatively rare subpopulation and so it is still possible that reduction in B cell progenitor numbers in *Wbp1l* ^−/−^ mice is caused by some aspect of CXCR4 signalling, which we could not measure.

WBP1L may also have other functions besides regulation of CXCR4. They may be responsible for a part of the phenotype of WBP1L‐deficient cells and animals. WBP1L binds multiple NEDD4 family members and very likely other proteins, which can result in pleiotropic effects on leucocyte biology. It has been shown that in competitive transplantation assay HSPC with only one functional CXCR4 allele perform better than wild‐type cells, which perform better than *CXCR4^WHIM^* cells, clearly showing an inverse correlation between CXCR4 activity and transplantation efficiency.^24^, ^25^ This observation is rather counterintuitive and opposite to the results we obtained with *Wbp1l* ^−/−^ cells. An explanation suggested in these studies was that CXCR4 promotes haematopoietic stem cell quiescence leading to competitive disadvantage when CXCR4 signalling is up‐regulated. This leads to the conclusion that though CXCR4 role cannot be completely excluded by our experiments, effects of WBP1L on transplantation efficiency are likely CXCR4‐independent. The molecular mechanism of how WBP1L regulates bone marrow engraftment will have to be addressed in future studies. On the other hand, functional effects of acute down‐regulation of WBP1L are more clearly connected to CXCR4, leading to increased surface expression of CXCR4, increased CXCR4 signalling and improved homing efficiency, similar to mouse models with increased CXCR4 activity.

It is at present unclear what is the reason for the unequal effects of the acute and constitutive OPAL1 deletion on CXCR4 signalling. We can speculate that WBP1L may be rather general regulator of expression and/or activity of NEDD4 family ligases. However, there are many additional mechanisms regulating these enzymes. In the majority of cases, these might be able to compensate for the loss of WBP1L in the long‐term. However, their ability to rapidly react to acute WBP1L loss would likely be more limited, as it may require changes in the gene expression pattern or other time‐consuming adaptations.

Another important question is the role that WBP1L potentially plays in leukaemia. The *WBP1L* gene is a target gene of ETV6, which suppresses its expression.^4^ In *ETV6‐RUNX1* ^+^ BCP‐ALL, one allele of *ETV6* is inactivated by fusion with *RUNX1*, while the other is often inactivated as well.^26^ This could explain the increase of WBP1L expression in *ETV6‐RUNX1* ^+^ BCP‐ALL. It is also in agreement with our data showing that in REH cells (which already have both *ETV6* alleles inactivated) *ETV6‐RUNX1* genetic deletion did not have any further effect on WBP1L expression. *ETV6* inactivation likely represents part of the mechanism leading to the development of leukaemia and defining its features. In principle, WBP1L in this context can have two different functions. First, negative regulation of CXCR4 by WBP1L could dampen the interactions of leukaemic (stem) cells with protective bone marrow niches, making them more sensitive to treatment. Second, WBP1L may also have negative effect on leukaemic stem cells similar to its negative regulatory effects on HSPCs that were revealed in our competitive transplantation experiment. Our data do not specifically address the role of WBP1L in leukaemia. However, the effects of WBP1L deficiency on normal haematopoiesis that we observed here and the fact that WBP1L is an ETV6 target gene make WBP1L a relevant target of future research in this field.

## CONFLICT OF INTEREST

The authors confirm that there are no conflicts of interest.

## AUTHOR CONTRIBUTIONS

SB, A.D and JK with contribution from MF, PA, JP, TS, NP and TB conducted majority of experiments and data analysis. DG performed microscopy analysis. IS carried out analysis of *Wbp1l* ^−/−^ embryos. OVM and KK analysed palmitoylation of WBP1L. V. Kanderova provided data on WBP1L expression in B cell lines. JS and PS generated ETV6‐RUNX1‐deficient REH cell line. MBP and MGT generated rabbit antisera to WBP1L. MAJ contributed to design and analysis of homing and competitive transplantation assays. V. Korinek contributed to the generation of *Wbp1l*‐CreERT mouse strain. TB with contribution from SB and AD conceptualized the study, evaluated the data and wrote the paper.

## Data Availability

The data that support the findings of this study are available from the corresponding author upon reasonable request.
