# Supplementary material for: Transmembrane adaptor protein WBP1L regulates CXCR4 signalling and murine haematopoiesis
Source: J Cell Mol Med. 2019 Dec 17;24(2):1980–92. doi: 10.1111/jcmm.14895 (PMC6991692; doi:10.1111/jcmm.14895)
Supplement: Supplementary file 1 [file JCMM-24-1980-s001.pdf]

Supplemental methods

Cell culture

REH cells were cultured in RPMI (Thermo Fisher Scientific, Waltham, MA), 293T and J774 cells and Phoenix cells were cultured in DMEM (Thermo Fisher), other BCP-ALL cell lines were cultivated as described previously [1]. Media were supplemented with 10% fetal bovine serum (Thermo Fisher) and antibiotics. Immortalized monocyte-macrophage progenitors were cultivated and generated using conditional HOXB8 construct as described previously [2,3]. KIT^+ cells were cultured in IMDM media supplemented with 0.1% IL-6, 0.2% IL-3, 1% SCF, supplied as supernatants from HEK293 cells transfected with constructs coding for respective cytokines. CRE-mediated *Wbp1l* deletion was induced with 1µM 4-hydroxytamoxifen. BMDM were prepared and cultivated as described [4].

Antibody generation

Preparation of the rabbit anti-WBP1L polyclonal antibody was outsourced to Eurogentec (Seraing, Belgium). The QAREHGHPHLPRPPAC peptide, which is close to the WBP1L C-terminus and is identical in the human and mouse sequences, was conjugated to keyhole limpet hemocyanin (KLH) for use as the antigen. The same peptide was used to affinity purify the antibody. OPAL-01 and OPAL-02 antibodies to human WBP1L were described previously [1]. mOPAL-01 and mOPAL-03 mouse monoclonal antibodies to murine WBP1L were generated by standard hybridoma technology using splenocytes of mice (F1 hybrids of BALB/c × B10) immunized with recombinant intracellular part of murine WBP1L protein and Sp2/0 myeloma cells as a fusion partner.

Flow cytometry

For surface staining, cells were incubated with antibodies and Fc-block (Clone 2.4G2) in PBS with 2% FBS for 30 min. For intracellular staining, cells were fixed with 4% formaldehyde (Thermo Fisher), then stained for surface markers, permeabilized with 90% methanol (Lachner, Neratovice, Czechia), blocked with 5% BSA (Merck, Darmstadt, Germany) 0.3% Triton X-100 (Merck) in PBS and stained for intracellular antigens in 1% BSA 0,3% Triton X-100 in PBS. For cell cycle analyses or Ki67 staining, cells were not permeabilized by methanol and after antibody staining were stained with 2 µg/ml Hoechst 33342 (Merck). Data were collected on LSRII flow cytometer (BD Biosciences, San Jose, CA) and analyzed with FlowJo software (FlowJo LLC, Ashland, OR).

Other reagents and their sources

β-estradiol (Merck), 4-Hydroxytamoxifen (Merck), recombinant murine and human SDF1α (Immunotools, Friesoythe, Germany), Lipofectamine 2000 (Thermo Fisher), Protein A/G (Santa Cruz Biotechnology, Dallas, TX), tunicamycin, Lysotracker Red DND-99, ER-Tracker Red, MitoTracker Red (Thermo Fisher), TC14012 (RanD corporation), AMD3100 (Merck).

Analysis of WBP1L palmitoylation

Palmitoylation of WBP1L was analyzed using click reaction-based approach, essentially as described earlier [5]. Briefly, HEK293 cells (5.5 x 10^5 in 60-mm dish) were transfected with 6 µg of WBP1L-FLAG-STREP-bearing pBABE using FuGENE (Promega, Madison, WI) according to manufacturer's instruction. After 24 h, cells were labeled with 50 µM 17-octadecynoic acid (17ODYA) or exposed to 0.05% DMSO carrier (1.5 h, 37 °C). Cells were lysed in a buffer containing 4% SDS and lysates subjected to click reaction with biotin-azide (500 µM biotin-PEG3-azide; Merck). Proteins were precipitated with chloroform:methanol [6] and resupended in the presence of 0.5% SDS and 1% Brij97. Biotin-tagged proteins were enriched on streptavidin-coupled beads (Thermo Fisher), eluted in hot H_2O followed by series of buffers and analyzed by immunoblotting using mouse anti-FLAG IgG (Merck) and rabbit anti-FLOTILLIN-2 IgG (Cell Signaling Technology, Danvers, MA).

Microscopy

BMDM were plated overnight on eight well µ-plate (IBIDi). The next day the cells were directly imaged or stained with organelle specific dye (MitoTracker Red 1:500, LysoTracker Red DND-99 1:1000, ER-tracker Red 1:1000, all Thermo Fisher) and directly imaged, or stained with CD11b-APC (Table S1) and subsequently fixed with 4% formaldehyde (Merck) and stained with Hoechst 33342 (Merck). Sequential 2-color imaging was performed using Leica TCS SP8 laser scanning confocal microscope with a 63×1.4 NA oil-immersion objective. Acquired images were manually thresholded to remove signal noise detected outside of the cell using ImageJ software.

Isolation of cell subsets from organs

Mouse blood was collected by cheek bleeding to EDTA tubes (KABE Labortechnik, Numbrecht-Elsenroth, Germany). B cells and T cells were isolated from splenocyte suspensions and neutrophils and KIT^+ cells from bone marrow cell suspensions using magnetic microbeads (Miltenyi Biotec, Bergisch Gladbach, Germany, see *Supplementary Table S5*) on an AutoMACS magnetic cell sorter (Miltenyi Biotec). Cells from murine embryo proper and yolk sac were isolated by digestion of respective tissue in dispase (1mg/ml, Thermo Fisher) in HBSS for 10 min followed by erythrocyte lysis with ACK buffer.

Knock-out cell line preparation

ETV6-RUNX1$^{-/-}$ REH cell lines were established using CRISPR/Cas9 technology. pLV-U6g-EPCG plasmid (Merck) was used with guide RNA (GTGCCTCGAGCGCTCAGGATGG) against exon 2 of *ETV6* gene. Since the second allele of *ETV6* is deleted in REH cell line, this targeting sequence is specific for fusion gene only. Knockout of *ETV6-RUNX1* gene was confirmed by Sanger sequencing, on mRNA and protein level (Figure S2 and data not shown). As a control, REH cell line transduced with non-targeting CRISPR/Cas9 vector was used.

Tandem purification of WBP1L for mass spectrometry analysis of WBP1L-binding proteins

Cells were lysed in lysis buffer (30mM TRIS, pH 7.4, 120 mM NaCl, 2 mM KCl, 10% Glycerol, 1% β-D-dodecylmaltoside, 10 mM Chloroacetamide, Phosphatase inhibitor tablets (PhosSTOP, Roche, Basel, Switzerland), Protease Inhibitor Cocktail (Roche)) and tagged proteins were immunoprecipated from postnuclear supernatants on anti-flag M2 affinity gel (Merck), eluted with 3x FLAG peptide (Merck), followed by second round of affinity purification on Strep-Tactin sepharose (IBA Lifesciences, Goettingen, Germany) and elution with 2% sodium deoxycholate (Merck) in 50mM TRIS (pH 8.5). Cysteines in eluted proteins were reduced with 5mM final concentration of TCEP (Tris(2-carboxyethyl)phosphine hydrochloride) and blocked with 10mM final concentration of MMTS (methyl methanethiosulfonate). Samples were cleaved with 1µg of trypsin. After digestion, samples were acidified with TFA (Trifluoroacetic acid) to 1% final concentration. Sodium deoxycholate was removed by extraction to ethylacetate [7]. Peptides were desalted on Michrom C18 column.

nLC-MS 2 Analysis

Nano Reversed phase column (EASY-Spray column, 50 cm x 75 µm ID, PepMap C18, 2 µm particles, 100 Å pore size) was used for LC/MS analysis. Mobile phase buffer A was composed of water and 0.1% formic acid. Mobile phase B was composed of acetonitrile and 0.1% formic acid. Samples were loaded onto the trap column (Acclaim PepMap300, C18, 5 µm, 300 Å Wide Pore, 300 µm x 5 mm, 5 Cartridges) for 4 min at 15 µl/min. Loading buffer was composed of water, 2% acetonitrile and 0.1% trifluoroacetic acid. Peptides were eluted with Mobile phase B gradient from 4% to 35% B in 60 min. Eluting peptide cations were converted to gas-phase ions by electrospray ionization and analyzed on a Thermo Orbitrap Fusion (Q-OT- qIT, Thermo Fisher). Survey scans of peptide precursors from 400 to 1600 m/z were performed at 120K resolution (at 200 m/z) with a 5×10^5 ion count target. Tandem MS was performed by isolation at 1.5 Th with the quadrupole, HCD (Higher-energy collisional dissociation) fragmentation with normalized collision energy of 30, and rapid scan MS analysis in the ion trap. The MS 2 ion count target was set to 10^4 and the max injection time was 35 ms. Only those precursors with charge state 2–6 were sampled for MS 2. The dynamic exclusion duration was set to 45 s with a 10 ppm tolerance around the selected precursor and its isotopes. Monoisotopic precursor selection was turned on. The instrument was run in top speed mode with 2 s cycles [8].

Data analysis of mass spectrometry

All data were analyzed and quantified with the MaxQuant software (version 1.5.3.8) [9]. The false discovery rate (FDR) was set to 1% for both proteins and peptides and we specified a minimum length of seven amino acids. The Andromeda search engine was used for the MS/MS spectra search against the *Mus musculus* database (downloaded from Uniprot on March 2018, containing 25 527 entries). Enzyme specificity was set as C-terminal to Arg and Lys, also allowing cleavage at proline bonds and a maximum of two missed cleavages. Dithiomethylation of cysteine was selected as fixed modification and N- terminal protein acetylation and methionine oxidation as variable modifications. The “match between runs” feature of MaxQuant was used to transfer identifications to other LC-MS/MS runs based on their masses and retention time (maximum deviation 0.7 min) and this was also used in quantification experiments. Quantifications were performed with the label-free algorithms described recently [9]. Data analysis was performed using Perseus 1.5.2.4 software.

Protein immunoprecipitation

Cells were lysed in lysis buffer described in the tandem purification method above. In co-Immunoprecipitation experiments 1% β-D-dodecylmaltoside was replaced with 1% NP-40 substitute (AppliChem GmbH) and in WBP1L immunoprecipitation from multiple B cell lines (Figure S2) RIPA buffer (50 mM TRIS pH7.4, 150 mM NaCl, 1% NP-40 substitute, 1% Deoxycholate (Merck), 0.1% SDS (Merck)) with the same protease and phosphatase inhibitors was used. Lysates were incubated with WBP1L monoclonal antibodies followed by isolation of antibody-bound complexes on protein A/G agarose beads (Santa Cruz Biotechnology) and elution with SDS-PAGE sample buffer.

Construct preparation and lentiviral production

If not otherwise specified, inserts were amplified from cDNA using Q5 polymerase (New England BioLabs, Boston, MA). Myc-tagged *WBP1L WT*, *ΔN* (with deletion of the following sequence QQRQHEINLIAYREAHNYSALPFYFRFLPNSLLPPYEEVVNRPPTPPPPYSAFQL deleted) *ΔC* (lacking entire C-terminus starting from PPPPQGGPPGGSPPGAD...) were generated by PCR and cloned into pcDNA3 vector. For WBP1L palmitoylation analysis and tandem purification, *WBP1L* or *EGFP* were cloned into tagging vector (pBABE containing C-terminal 3xFlag-2x Srep-tag-UGA –IRES-G418). For microscopy, *WBP1L* was cloned into MSCV vector in front of *MYC-tag-EGFP-UAA*. *Golgi-mApple* was amplified from mApple-Golgi-7 (mApple-Golgi-7 was a gift from Michael Davidson, Addgene plasmid # 54907) and subcloned into MSCV. E3 ligase cloning is summarized in Table S3. *WBP1L* shRNA (silencing/nonsilencing murine TRCN0000297606/085, human TRCN0000282025/275362) was obtained from Merck. Full length *Cre* (a gift from Dušan Hrčkulák) was cloned into pHIV-EGFP vector (pHIV-EGFP was a gift from Bryan Welm & Zena Werb, Addgene plasmid # 21373 [10]).

For lentiviral transductions HEK293T cells were transfected with Lentiviral Packaging vector (Thermo Fisher) and a vector of interest in ratio 1:2.5 using polyethyleneimine (PEI) (Polysciences, Warrington, PA). Virus was concentrated on centrifugal filter (Amicon 100K, Millipore). Cell infection was performed similarly to retroviruses as described here [4]. After infection with shRNA constructs, the infected cells were sorted based on the reporter (EGFP) expression and used in experiments no later than 3 weeks after the infection.

Table S1. List of flow cytometry antibodies

Antigen	Clone	Species tested	Conjugate	Company
P-ERK	197G	Mus musculus	Alexa 647	Cell Signalling Technology
Ly5.1	A20	Mus musculus	APC, FITC	BioLegend
Ly5.2	104	Mus musculus	PE-Cy7, PB,	BioLegend
CD3	17A2, 1452/C11	Mus musculus	PE, PB	BioLegend
Ly6C	HK1.4	Mus musculus	FITC, PE-Cy7	BioLegend
Ly6G	1A8	Mus musculus	PB, FITC, PB, APC	BioLegend
B220	RA3-6B2	Mus musculus	e450	Thermo-Fisher
IgM	EB121-15F9	Mus musculus	FITC	eBioscience
CD43	eBioR2/60	Mus musculus	PE	eBioscience
KIT	2B8	Mus musculus	PE, FITC	BioLegend
SCA-1	E13-161.7	Mus musculus	APC	BioLegend
CD16/32	93	Mus musculus	FITC	BioLegend
CD19	6D5	Mus musculus	FITC	BioLegend
CD11c	N418	Mus musculus	APC	BioLegend
F4/80	BM8	Mus musculus	PE, FITC, PE-Cy7	BioLegend
CD11b	M1/70	Mus musculus	PE, BV785, A700, APC	BioLegend, Sony
TER119	TER-119	Mus musculus	PB, Qdot605	BioLegend,
CD34	RAM34	Mus musculus	FITC	eBioscience
CD93	AA4.1	Mus musculus	PerCP-Cy5.5	BioLegend
CD23	B3B4	Mus musculus	e660, APC	Thermo-Fisher
CD1d	1B1	Mus musculus	FITC	BioLegend
CD5	57-7.3	Mus musculus	PE	Thermo-Fisher
CD45	30-F11	Mus musculus	PerCP-Cy5.5	Biolegend
Ki67	16A8	Mus musculus	APC	Biolegend
anti-mouse Lineage Cocktail	17A2/RB6-8C5/RA3-6B2/Ter-119/M1/70	Mus musculus	PB	BioLegend
KOMBITEST™ CD3 FITC / CD16+ CD56 + PE / CD45 PerCP / CD19 APC	UCHT1/3G8/LT56/MEM-28/LT19	Homo sapiens	FITC, PE, PerCP, APC	Exbio
CD14	MEM-18	Homo sapiens	FITC	Exbio
CXCR4	2B11	Mus musculus and Homo sapiens	APC	Thermo-Fisher

Table S2. List of antibodies for immunoprecipitation and Western blotting.

Antigen	Clone	Company	Comments
WBP1L	Rabbit polyclonal	Custom made, Eurogentec	Human/mouse WBP1L for Western Blotting
WBP1L	OPAL-01	Made in house	Human WBP1L
WBP1L	OPAL-02	Made in house	Human WBP1L
WBP1L	mOPAL-01	Made in house	Mouse WBP1L
WBP1L	mOPAL-03	Made in house	Mouse WBP1L
β-ACTIN	AC15	Merck	
P-ERK	197G2	Cell Signalling Technology	
ERK 1	MK12	BD Bioscience	
ERK 2	Rabbit polyclonal	Santa Cruz Biotechnology	
P-AKT	D9E	Cell Signalling Technology	
GAPDH	Rabbit polyclonal	Merck	
MYC-tag	9B11	Cell Signalling Technology	
UBIQUITIN	Rabbit polyclonal (A100)	Boston Biochem	
UBIQUITIN	P4D1	Cell Signalling Technology	CXCR4 ubiquitination
ITCH	Rabbit polyclonal	LifeSpan BioSciences	Itch staining in Fig. 5C
ITCH	D8Q6D	Cell Signalling Technology	Itch expression in progenitors
WWP1	Rabbit polyclonal	Abcam	
WWP2	Rabbit polyclonal	Abcam	
CXCR4	2B11	BD Bioscience	
HA-tag	C29F4	Cell Signalling Technology	
FLOTILLIN-2	Rabbit monoclonal	Cell Signalling Technology	Western Blotting
FLAG	M2	Merck	
Mouse Anti-Rabbit IgG Antibody conjugated to peroxidase	M205	Genscript	For I.P.
Goat Anti-Mouse, light chain specific, conjugated to peroxidase	Monoclonal, not specified	Jackson Immunoresearch	For I.P.
Mouse Anti-Rabbit, light chain specific, conjugated to peroxidase	Monoclonal, not specified	Jackson Immunoresearch	For I.P.
Goat anti-Mouse IgG (H+L), conjugated to peroxidase	Polyclonal	Bio-Rad	For lysates
Goat anti-Mouse IgG (H+L), conjugated to peroxidase	Polyclonal	Bio-Rad	For lysates

Table S3. List of qPCR primers

Primer	Sequence	Species tested	Company
WBP1L forward	CTCAGCGCTGCCATTTTATT	Homo sapiens	Merck
WBP1L reverse	GCTGGAAGGCACTGTATGGT	Homo sapiens	Merck
GAPDH forward	CCACATCGCTCAGACACCAT	Homo sapiens	Merck
GAPDH reverse	CCAGGCGCCCAATACG	Homo sapiens	Merck
WBP1L forward	CGTTGCCGTTTTACTTCAGG	Mus musculus	Merck
WBP1L reverse	GAGCTGGAAGGCACTGTACG	Mus musculus	Merck
WWP1 forward	GTTGCTGCCAGACCCAAA	Mus musculus	Merck
WWP1 reverse	TAGGACAGATGATGATTCTCCATTA	Mus musculus	Merck
WWP2 forward	GCCGGTTACCAGCTCAAA	Mus musculus	Merck
WWP2 reverse	TCAAAGATACAGGTCTGCAAGC	Mus musculus	Merck
SMURF2 forward	TTACATGAGCAGGACACACTTACA	Mus musculus	Merck
SMURF2 reverse	GCTGCGTTGTCCTTTGTTC	Mus musculus	Merck
SMURF1 forward	GGGTCAGTGGTGGACTGC	Mus musculus	Merck
SMURF1 reverse	CCAGGGCCTGAGTCTTCATA	Mus musculus	Merck
NEDD4L forward	TGAGCAAGCTCACCTTCCA	Mus musculus	Merck
NEDD4L reverse	CCCGTGACAGTTGACGAAC	Mus musculus	Merck
NEDD4 forward	GCCGGTTACCAGCTCAAA	Mus musculus	Merck
NEDD4 reverse	TCAAAGATACAGGTCTGCAAGC	Mus musculus	Merck
ITCH forward	TTGATGCGAAGGAATTAGAGG	Mus musculus	Merck
ITCH reverse	GGTGTAGTGGCGGTAGATGG	Mus musculus	Merck
β-ACTIN forward	GATCTGGCACCACACCTTCT	Mus musculus	Merck
β-ACTIN reverse	GGGGTGTTGAAGGTCTCAAA	Mus musculus	Merck

Table S4. List of Nedd4-family cDNA constructs.

E3 ligase	Species	Plasmid or insert origin	Recloned to pK-MYC-C1 and thus adding Myc-tag
NEDD4L	Homo sapiens	Addgene, Plasmid: 27000<br>Gift from Joan Massague [11]	+
ITCH	Mus musculus	Addgene, Plasmid 11427<br>Gift from Allan Weissman [12]	-
WWP1	Homo sapiens	Gift from Paul Bieniasz, ADARC, NY	+
WWP2	Homo sapiens	Gift from Paul Bieniasz, ADARC, NY	+

Table S5 List of magnetic cell sorting reagents.

Antigen	Clone	Species tested	Conjugate	Company
Ly6G	17A2	Mus musculus	Biotin	BioLegend
CD3	145/2C11	Mus musculus	Biotin	BioLegend
CD11b MicroBeads	M1/70	Mus musculus and Homo sapiens	Coated	MiltenyiBiotec
CD43 (Ly48) MicroBeads	not specified by the company	Mus musculus	Coated	MiltenyiBiotec
KIT	Ack2	Mus musculus	Biotin	In house

Supplemental figures

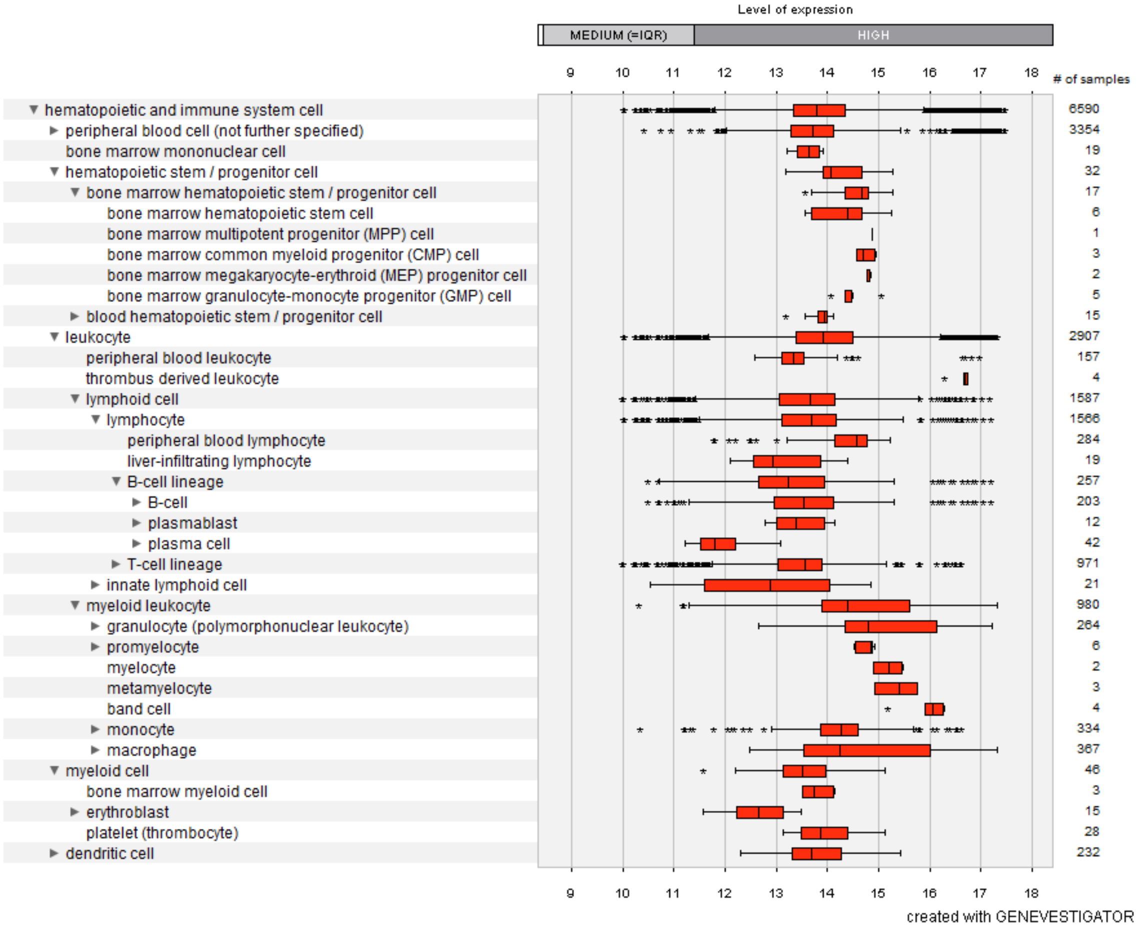

Figure S1. *WBP1L* expression in human leukocytes and leukocyte progenitor subsets. Expression profile of *WBP1L* mRNA generated by Genevestigator gene expression analysis tool, based on manually curated gene expression data from public repositories [13]. The box delimits the area between the upper and lower quartiles. Whiskers represent the lowest or highest data point still within 1.5 times this area in each direction. Stars represent data points outside this range.

A

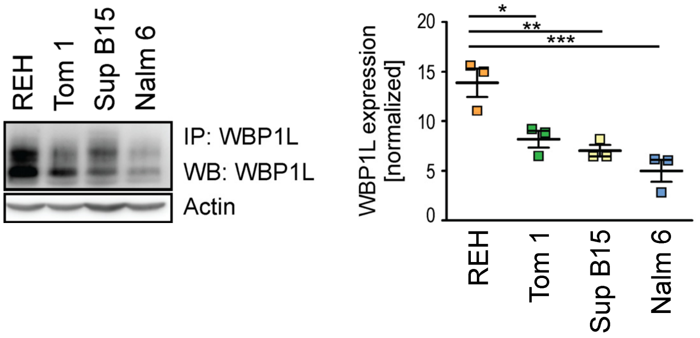

B

```
Original sequence   150 GCCTCGAGCGTCA--------GGATGGAGGAAGACTCG 180
Clone #17               .............AA------.................
Clone #23               .............CG------.................
Clone #190              .............GG------.................
Clone #192              .............GGGGC---.................
Clone #200              .............AGATAGAC.................
```

C

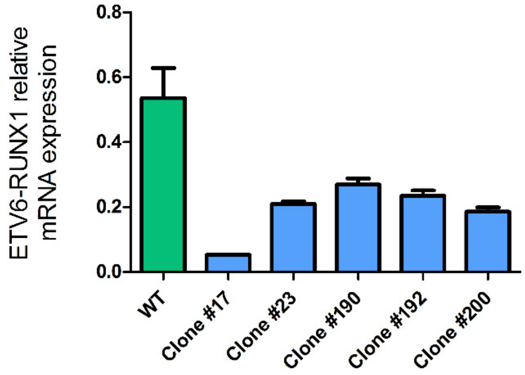

Figure S2. Expression of WBP1L in ETV6-RUNX1^{+} and ETV6-RUNX1^{-} cell lines and deletion of ETV6-RUNX1 in REH cells. (A) WBP1L immunoprecipitates from *ETV6-RUNX1^{+}* B cell line REH and *ETV6-RUNX1^{-}* lines TOM-1, NALM-6, and SUB B15 were immunoblotted with antibody to WBP1L and actin. For quantification, data were normalized in each experiment to actin and then to experiment average to allow comparison among experiments. Statistical significance was calculated using one way ANOVA with Dunnett's post test. (N=3). **(B)** Results of *ETV6* exon2 CRISPR/CAS9 target site sequencing in individual *ETV6-RUNX1*-deficient REH clones. **(C)** *ETV6-RUNX1* mRNA expression in the clones from (B) determined by qPCR. The data are plotted as $2^{-\Delta CT}$.

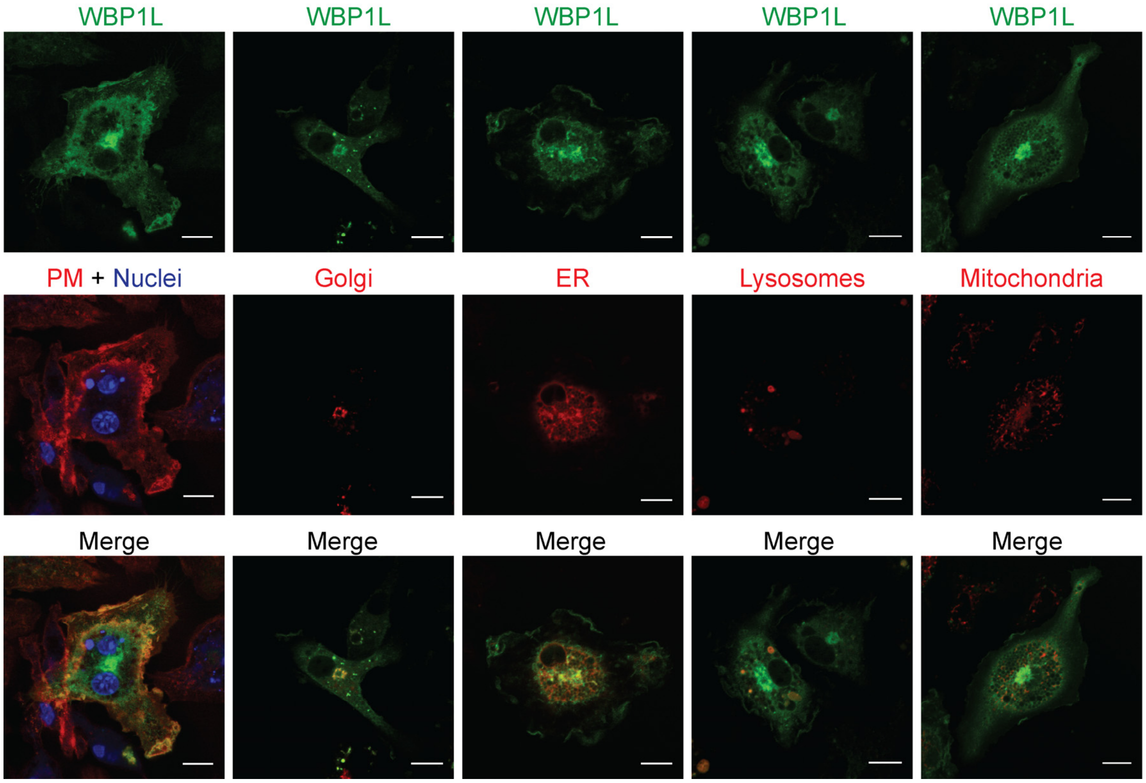

Figure S3. Subcellular localization of WBP1L. Confocal imaging of BMDM from *Wbp1l*$^{-/-}$ mice transduced with WBP1L-EGFP. The following markers were used: Plasma membrane (PM) – CD11b, Nuclei – Hoechst 33342, both on fixed cells; Golgi – Golgi-7-mApple retroviral construct, Endoplasmic reticulum (ER) – ER-Tracker-Red, Lysosomes and other acidic organelles – LysoTracker Red, Mitochondria – MitoTracker Red, all live cell imaging. Bar = 10 µm. N ≥ 3.

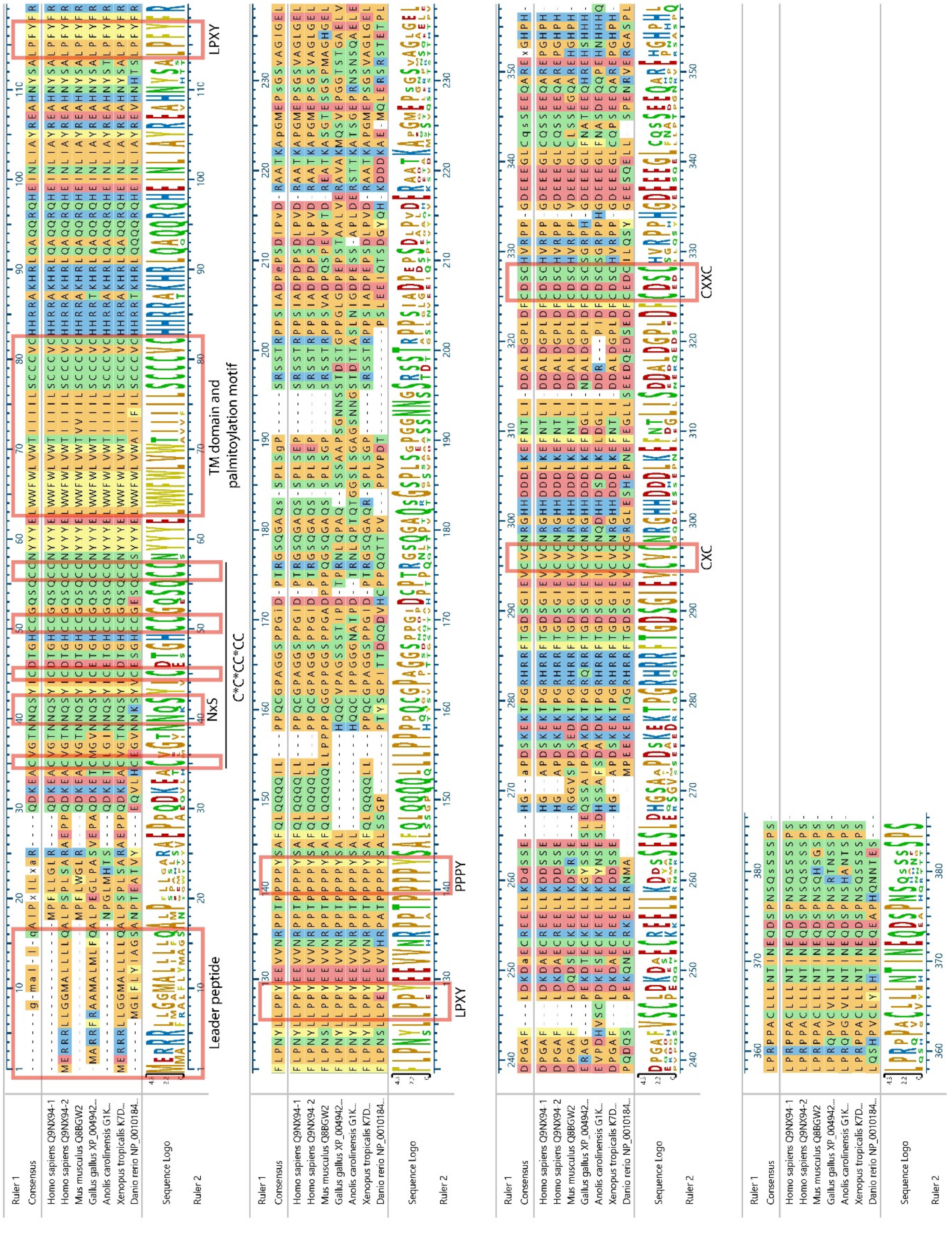

Figure S4. Alignment of WBP1L sequences from representatives of major vertebrate classes. Sequences were aligned with MUSCLE algorithm using MegAlign Pro software from DNASTAR Lasegene suite. Conserved features are highlighted with red boxes.

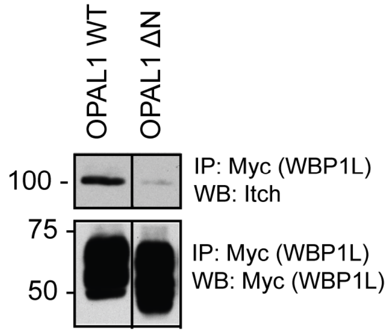

Figure S5. WBP1L interaction with ITCH in J774 macrophage-like cell line. MYC-tag imunoprecipatates from J774 cells stably expressing MYC-tagged WBP1L wild-type form or WBP1LΔN lacking WW domain interacting motifs. Western blots were stained with anti-ITCH antibody and anti MYC-tag antibody. Irrelevant lines from the blot image were removed and replaced with vertical dividing lines. N=2.

A

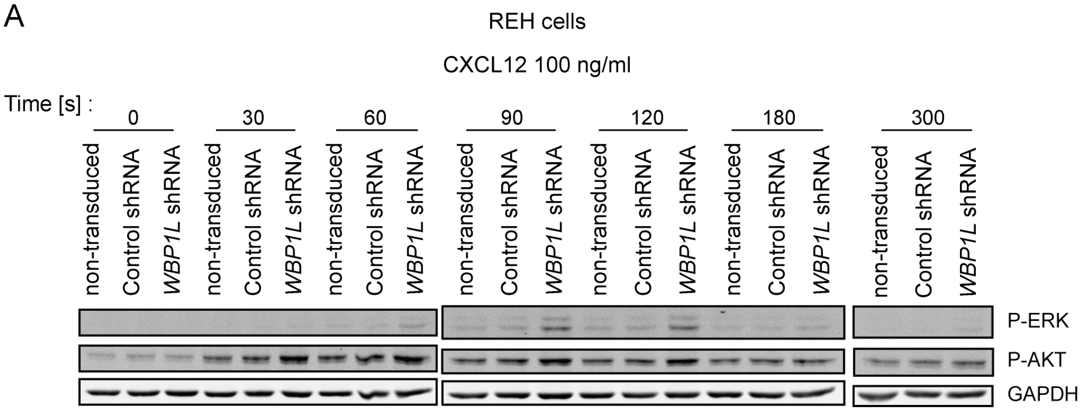

B

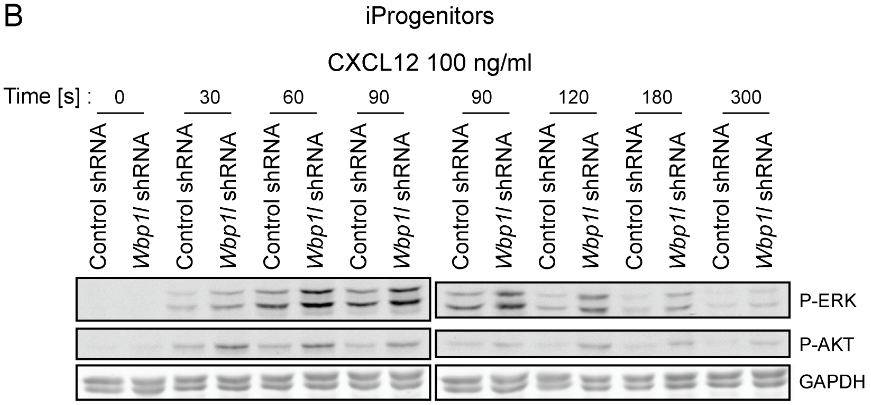

Figure S6. Representative Western blots showing data quantified in Figure 4C (A) and 4D (B).

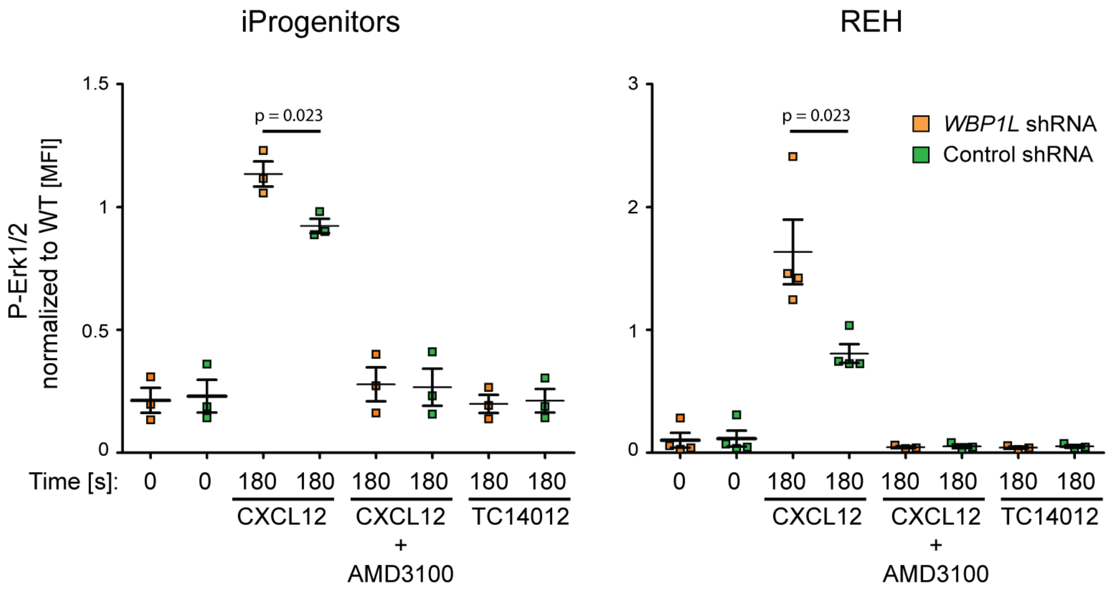

Figure S7. ERK1/2 phosphorylation downstream of CXCR4 or CXCR7 in immortalized monocyte/macrophage progenitors and REH cells transduced with *WBP1L* or control shRNA. Cells were treated with 100 nM CXCL12 alone or together with 1 µg/ml of CXCR4 antagonist AMD3100 or treated with 1 µM specific agonist of CXCR7 TC14012. ERK1/2 phosphorylation was analyzed by flow cytometry. Data are normalized to non-transduced cells (left panel N=3, right panel N=4).

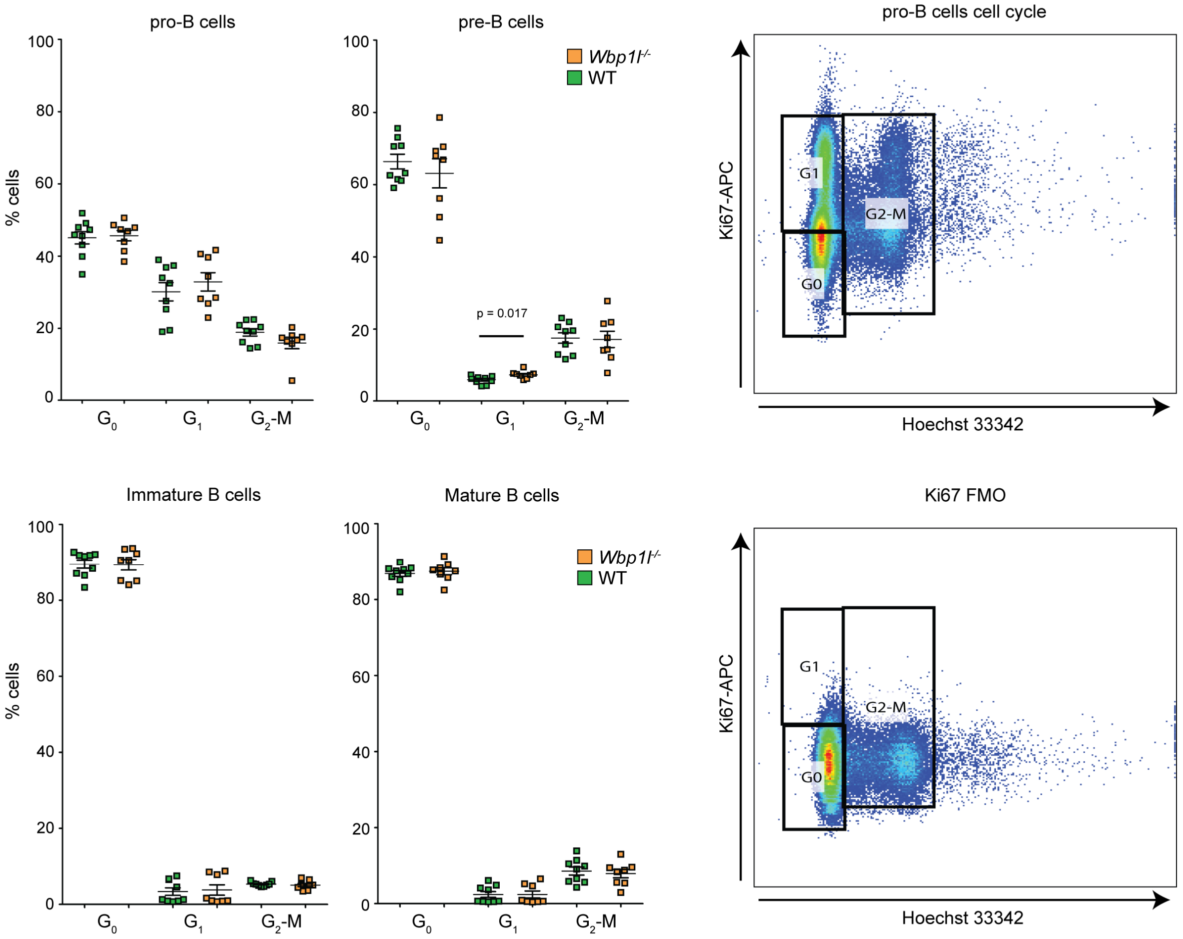

Figure S8. Analysis of cell cycle in bone marrow B cell progenitors from wild-type and *Wbp1l*-deficient mice. Bone marrow cells were fixed, stained for surface markers, permeabilized, and stained with Ki67 and Hoechst 33342. Individual B cell subsets were defined using following markers: Pro-B cells ($CD43^{+}$, $B220^{+}$, IgM^{-}), Pre-B cells ($CD43^{-}$, $B220^{low}$, IgM^{-}), Immature B cells ($CD43^{-}$, $B220^{low}$, IgM^{+}), Mature B cells ($CD43^{-}$, $B220^{high}$, IgM^{+}). The right panel of FACS plots shows gating strategy of cell cycle analyses. Representative FACS plots of stained cells with and without (Ki67 FMO) Ki67 staining are shown.

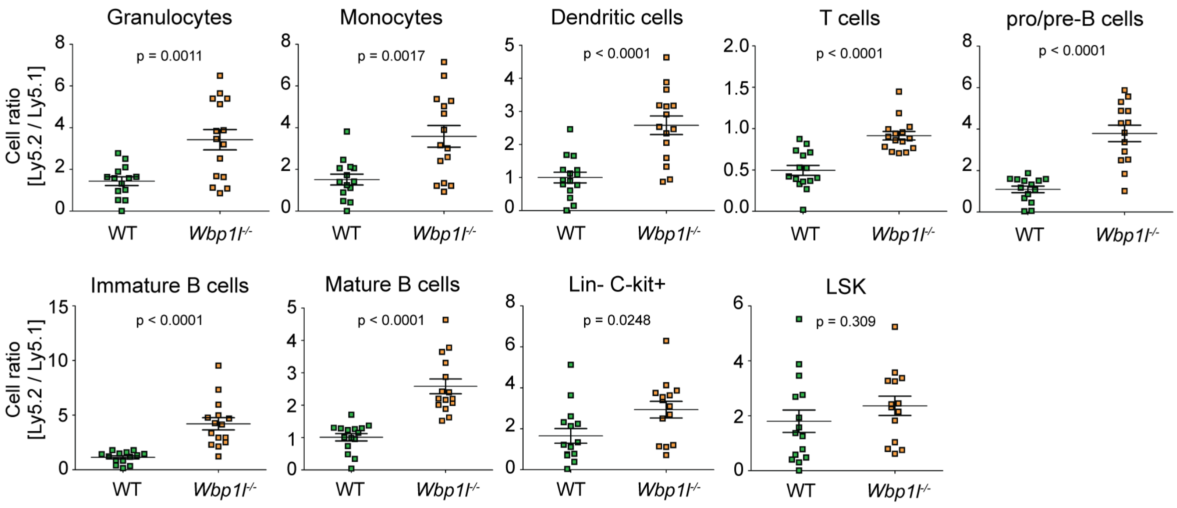

Figure S9. Enhanced engraftment of *Wbp1l*$^{-/-}$ bone marrow. Ly5.2^{+} bone marrow (WT or *Wbp1l*$^{-/-}$) was mixed with Ly5.1^{+} bone marrow (always WT) in a ratio 1:1 and 2×10^{6} cells were transplanted into Ly5.1 lethally irradiated mice. Mice were analyzed two months post transplantation. Flow cytometry analyses show the ratio between Ly5.2 and Ly5.1 cells determined for individual bone marrow cell subsets. Individual cell subsets were defined using following markers: Granulocytes (LY6C^{-}, CD11b^{+}, LY6G^{+}), Monocytes (LY6C^{+}, CD11b^{+}, LY6G^{-}), Dendritic cells (CD11c^{+}), T cells (CD3^{+}), Pro/Pre B cells (B220^{low}, IgM^{-}), Immature B cells (B220^{low}, IgM^{+}), Mature B cells (B220^{high}, IgM^{+}), Lin^{-}C-kit^{+} cells (lin^{-}, c-kit^{+}), LSK cells (lin^{-}, c-kit^{+}, SCA1^{+}).

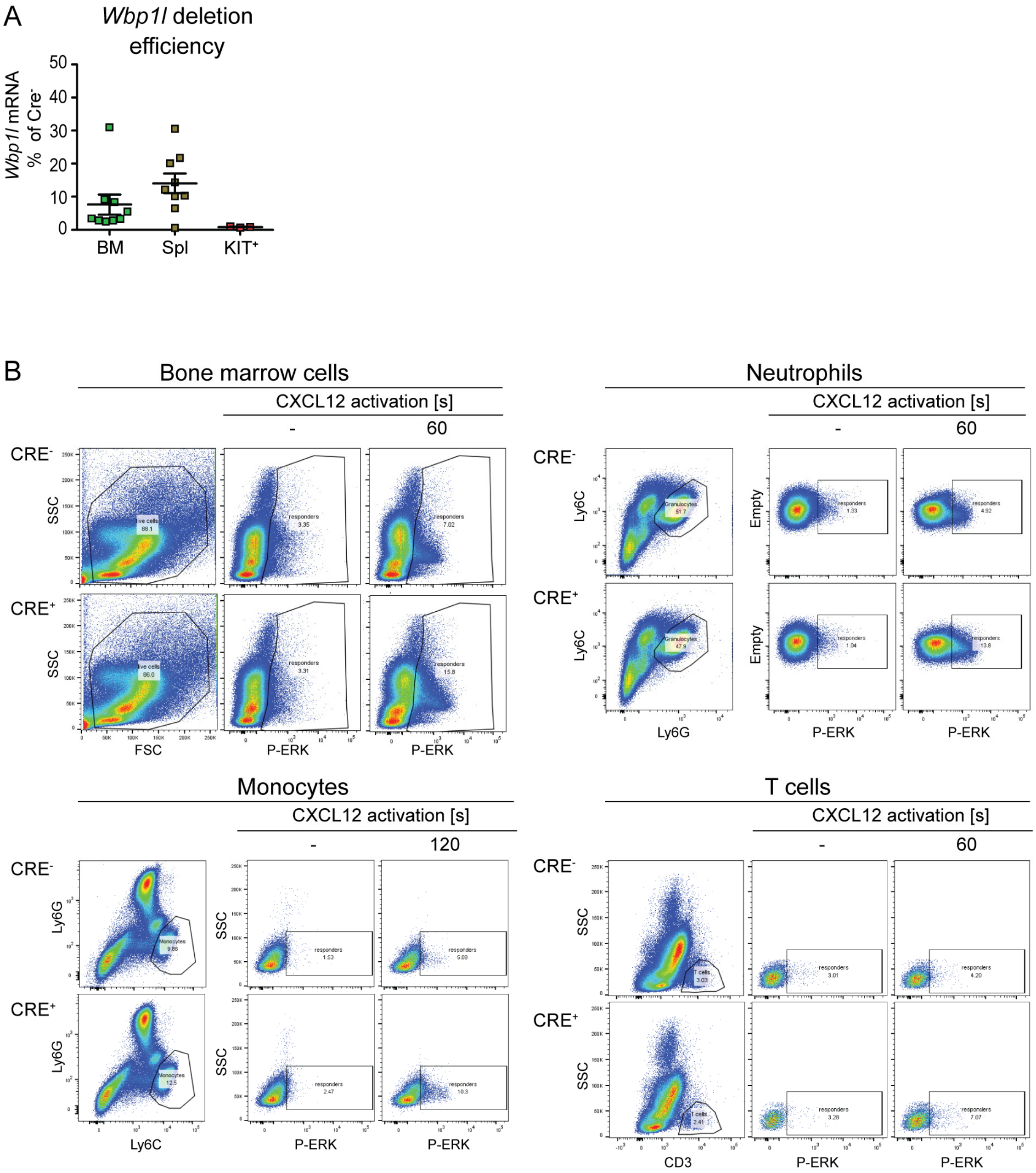

Figure S10. Effects of inducible *Wbp1l* deletion on ERK activation in bone marrow cell subsets. (A) *Wbp1l* mRNA expression after in vitro 4-hydroxytamoxifen induced deletion in samples from Figure 7F compared to 4-hydroxytamoxifen-induced deletion in vivo in the whole bone marrow and spleen from *Wbp1l-CreERT* mice (samples from Figure 7D). (B) Representative FACS plots for data shown in Figure 7D.

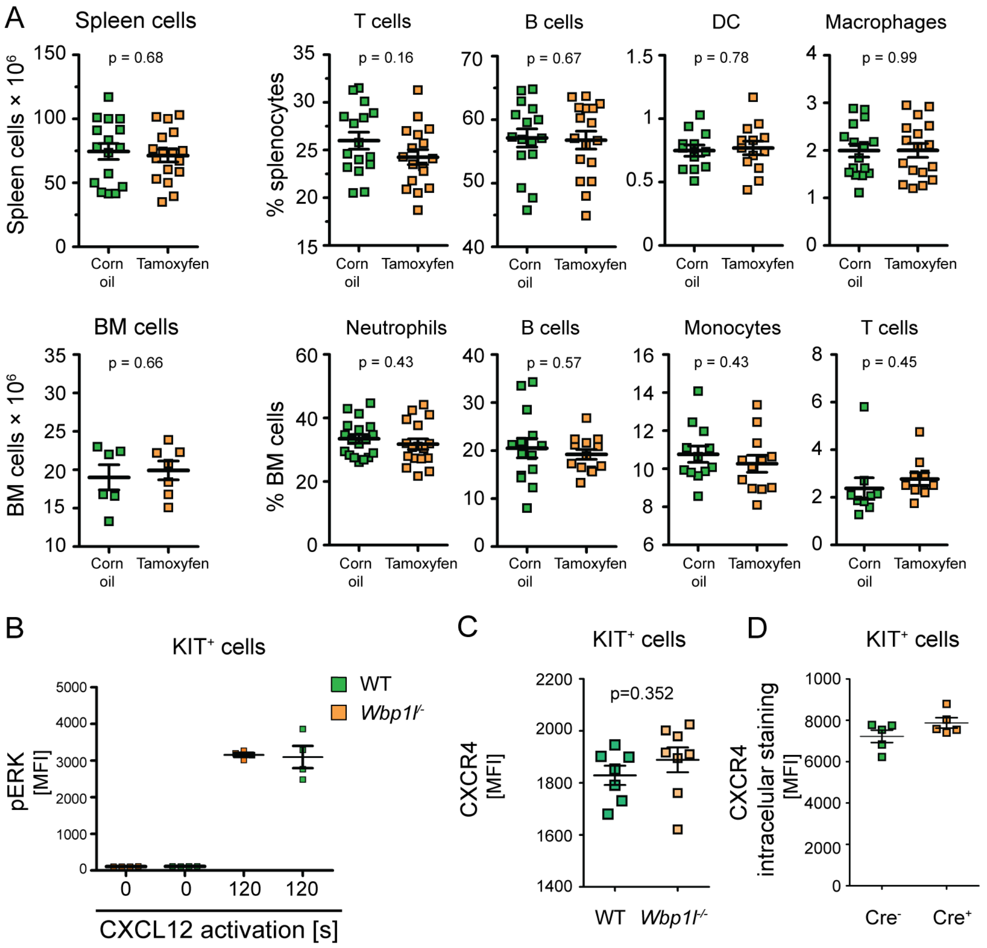

Figure S11. Additional analyses of *Wbp1l$^{-/-}$* cells. (A) Total numbers of splenocytes and bone marrow cells and percentages of major leukocyte subsets 5-7 days after Tamoxifen-induced deletion of *Wbp1l*. The splenocyte subsets were defined using the following markers: T cells (CD3$^+$), B cells (B220$^+$), DC (CD11c$^+$, Ly6C$^{-/low}$), Macrophages (F4/80$^+$, CD11b^{int}). The bone marrow cell subsets were defined using the following markers: Neutrophils (Ly6C^{int}, CD11b$^+$, Ly6G$^+$), B cells (B220$^+$), Monocytes (Ly6C^{high}, CD11b$^+$, Ly6G$^-$), T cells (CD3$^+$). (B) Similar experiment as in Figure 7F. However, here the cells with constitutive *Wbp1l* inactivation were used (N=4). (C) CXCR4 surface expression was measured in cultured KIT$^+$ bone marrow progenitors with constitutive *Wbp1l* inactivation by flow cytometry. One significant outlier was removed based on Q test. (D) Total CXCR4 expression was measured in cultured KIT$^+$ bone marrow progenitors with constitutive *Wbp1l* inactivation by flow cytometry of fixed and permeabilized cells.

Supplemental References

1. **Kanderova V, Kuzilkova D, Stuchly J, Vaskova M, Brdicka T, Fiser K, Hrusak O, Lund-Johansen F, Kalina T.** High-resolution Antibody Array Analysis of Childhood Acute Leukemia Cells. *Mol Cell Proteomics*. 2016; 15: 1246-61.
2. **Wang GG, Calvo KR, Pasillas MP, Sykes DB, Hacker H, Kamps MP.** Quantitative production of macrophages or neutrophils ex vivo using conditional Hoxb8. *Nat Methods*. 2006; 3: 287-93.
3. **Drobek A, Kralova J, Skopcova T, Kucova M, Novak P, Angelisova P, Otahal P, Alberich-Jorda M, Brdicka T.** PSTPIP2, a Protein Associated with Autoinflammatory Disease, Interacts with Inhibitory Enzymes SHIP1 and Csk. *J Immunol*. 2015; 195: 3416-26.
4. **Kralova J, Glatzova D, Borna S, Brdicka T.** Expression of Fluorescent Fusion Proteins in Murine Bone Marrow-Derived Dendritic Cells and Macrophages. *J Vis Exp*. 2018; 140: e58081.
5. **Sobocinska J, Roszczenko-Jasinska P, Zareba-Koziol M, Hromada-Judycka A, Matveichuk OV, Traczyk G, Lukasiuk K, Kwiatkowska K.** Lipopolysaccharide Upregulates Palmitoylated Enzymes of the Phosphatidylinositol Cycle: An Insight from Proteomic Studies. *Mol Cell Proteomics*. 2018; 17: 233-54.
6. **Wessel D, Flugge UI.** A method for the quantitative recovery of protein in dilute solution in the presence of detergents and lipids. *Anal Biochem*. 1984; 138: 141-3.
7. **Masuda T, Tomita M, Ishihama Y.** Phase transfer surfactant-aided trypsin digestion for membrane proteome analysis. *J Proteome Res*. 2008; 7: 731-40.
8. **Hebert AS, Richards AL, Bailey DJ, Ulbrich A, Coughlin EE, Westphall MS, Coon JJ.** The one hour yeast proteome. *Mol Cell Proteomics*. 2014; 13: 339-47.
9. **Cox J, Hein MY, Luber CA, Paron I, Nagaraj N, Mann M.** Accurate proteome-wide label-free quantification by delayed normalization and maximal peptide ratio extraction, termed MaxLFQ. *Mol Cell Proteomics*. 2014; 13: 2513-26.
10. **Welm BE, Dijkgraaf GJ, Bledau AS, Welm AL, Werb Z.** Lentiviral transduction of mammary stem cells for analysis of gene function during development and cancer. *Cell Stem Cell*. 2008; 2: 90-102.
11. **Gao S, Alarcon C, Sapkota G, Rahman S, Chen PY, Goerner N, Macias MJ, Erdjument-Bromage H, Tempst P, Massague J.** Ubiquitin ligase Nedd4L targets activated Smad2/3 to limit TGF-beta signaling. *Mol Cell*. 2009; 36: 457-68.
12. **Magnifico A, Ettenberg S, Yang C, Mariano J, Tiwari S, Fang S, Lipkowitz S, Weissman AM.** WW domain HECT E3s target Cbl RING finger E3s for proteasomal degradation. *J Biol Chem*. 2003; 278: 43169-77.
13. **Hruz T, Laule O, Szabo G, Wessendorp F, Bleuler S, Oertle L, Widmayer P, Gruissem W, Zimmermann P.** Genevestigator v3: a reference expression database for the meta-analysis of transcriptomes. *Adv Bioinformatics*. 2008; 2008: 420747.
